# Large-scale genome sequencing of mycorrhizal fungi provides insights into the early evolution of symbiotic traits

**DOI:** 10.1038/s41467-020-18795-w

**Published:** 2020-10-12

**Authors:** Shingo Miyauchi, Enikő Kiss, Alan Kuo, Elodie Drula, Annegret Kohler, Marisol Sánchez-García, Emmanuelle Morin, Bill Andreopoulos, Kerrie W. Barry, Gregory Bonito, Marc Buée, Akiko Carver, Cindy Chen, Nicolas Cichocki, Alicia Clum, David Culley, Pedro W. Crous, Laure Fauchery, Mariangela Girlanda, Richard D. Hayes, Zsófia Kéri, Kurt LaButti, Anna Lipzen, Vincent Lombard, Jon Magnuson, François Maillard, Claude Murat, Matt Nolan, Robin A. Ohm, Jasmyn Pangilinan, Maíra de Freitas Pereira, Silvia Perotto, Martina Peter, Stephanie Pfister, Robert Riley, Yaron Sitrit, J. Benjamin Stielow, Gergely Szöllősi, Lucia Žifčáková, Martina Štursová, Joseph W. Spatafora, Leho Tedersoo, Lu-Min Vaario, Akiyoshi Yamada, Mi Yan, Pengfei Wang, Jianping Xu, Tom Bruns, Petr Baldrian, Rytas Vilgalys, Christophe Dunand, Bernard Henrissat, Igor V. Grigoriev, David Hibbett, László G. Nagy, Francis M. Martin

**Affiliations:** 1 Université de Lorraine, Institut national de recherche pour l’agriculture, l’alimentation et l’ environnement, UMR Interactions Arbres/Microorganismes, Centre INRAE Grand Est-Nancy, 54280 Champenoux, France; 2 Synthetic and Systems Biology Unit, Biological Research Centre, 6726 Szeged, Hungary; 3 US Department of Energy Joint Genome Institute, Lawrence Berkeley National Laboratory, Berkeley, CA USA; 4 INRAE, USC1408 Architecture et Fonction des Macromolécules Biologiques, 13009 Marseille, France; 5 Biology Department, Clark University, Lasry Center for Bioscience, 950 Main Street, Worcester, MA 01610 USA; 6 Plant Soil and Microbial Sciences, Michigan State University, East Lansing, MI 48824 USA; 7 Chemical & Biological Processes Development Group, Pacific Northwest National Laboratory, Richland, WA USA; 8 Westerdijk Fungal Biodiversity Institute, Uppsalalaan 8, 3584 CT Utrecht, Netherlands; 9 Department of Life Sciences and Systems Biology, University of Torino, Viale Mattioli 25, 10125 Torino, Italy; 10 Swiss Federal Institute for Forest, Snow and Landscape Research WSL, Zuercherstrasse 111, 8903 Birmensdorf, Switzerland; 11 The Jacob Blaustein Institutes for Desert Research, Bergman Campus, Ben-Gurion University of The Negev, Beer-Sheva, Israel; 12 Laboratory of Environmental Microbiology, Institute of Microbiology of the Czech Academy of Sciences, Videnska 1083, 14220 Praha 4, Czech Republic; 13 Department Botany & Plant Pathology, Oregon State University, Corvallis, OR USA; 14 Natural History Museum, University of Tartu, 14a Ravila, 50411 Tartu, Estonia; 15 Department of Forest Sciences, University of Helsinki, Helsinki, Finland; 16 Institute of Mountain Science, Faculty of Agriculture, Shinshu University, Minami-minowa, Kami-ina, Nagano, 399-4598 Japan; 17 Department of Key Laboratory, The 2nd Affiliated Hospital of Kunming Medical University, 374 Dian Mian Road, Kunming, 650101 Yunnan China; 18 Department of Biology, McMaster University, 1280 Main St. West, Hamilton, ON L8S 4K1 Canada; 19 Department of Plant and Microbial Biology, University of California – Berkeley, Berkeley, CA USA; 20 Department of Biology, Duke University, Durham, NC 27708 USA; 21 Laboratoire de Recherche en Sciences Végétales, Université de Toulouse, CNRS, UPS, Toulouse, France; 22 Architecture et Fonction des Macromolécules Biologiques (AFMB), CNRS, Aix-Marseille Univ., 13009 Marseille, France; 23 Department of Biological Sciences, King Abdulaziz University, Jeddah, Saudi Arabia; 24 Beijing Advanced Innovation Centre for Tree Breeding by Molecular Design (BAIC-TBMD), Institute of Microbiology, Beijing Forestry University, Tsinghua East Road Haidian District, Beijing, China

**Keywords:** Microbial ecology, Evolutionary developmental biology

## Abstract

Mycorrhizal fungi are mutualists that play crucial roles in nutrient acquisition in terrestrial ecosystems. Mycorrhizal symbioses arose repeatedly across multiple lineages of Mucoromycotina, Ascomycota, and Basidiomycota. Considerable variation exists in the capacity of mycorrhizal fungi to acquire carbon from soil organic matter. Here, we present a combined analysis of 135 fungal genomes from 73 saprotrophic, endophytic and pathogenic species, and 62 mycorrhizal species, including 29 new mycorrhizal genomes. This study samples ecologically dominant fungal guilds for which there were previously no symbiotic genomes available, including ectomycorrhizal Russulales, Thelephorales and Cantharellales. Our analyses show that transitions from saprotrophy to symbiosis involve (1) widespread losses of degrading enzymes acting on lignin and cellulose, (2) co-option of genes present in saprotrophic ancestors to fulfill new symbiotic functions, (3) diversification of novel, lineage-specific symbiosis-induced genes, (4) proliferation of transposable elements and (5) divergent genetic innovations underlying the convergent origins of the ectomycorrhizal guild.

## Abstract (web summary)

Mycorrhizal symbioses have evolved repeatedly in diverse fungal lineages. A large phylogenomic analysis sheds light on genomic changes associated with transitions from saprotrophy to symbiosis, including divergent genetic innovations underlying the convergent origins of the ectomycorrhizal guild.

## Introduction

Mycorrhizal fungi are central to the evolution, biology, and physiology of land plants because they promote plant growth by facilitating the acquisition of scarce and essential nutrients, such as phosphorus and nitrogen^1–3^. They are also major drivers of carbon sequestration and they have a well-documented impact on the composition of microbial and plant communities^1,2^. The most ubiquitous classes of mycorrhizal symbioses are ectomycorrhiza, arbuscular mycorrhiza, orchid mycorrhiza, and ericoid mycorrhiza^4,5^. Each class is classified based on host plant and characteristic symbiotic structures. Although mycorrhizal fungi are highly diverse in terms of their evolutionary history, the independent evolution of similar symbiotic morphological structures, and physiological traits in divergent fungal taxa provides a striking example of convergent evolution^3–5^. Although unique and common traits in mycorrhizal symbioses have recently been reviewed^2^, molecular mechanisms underlying these convergent phenotypes remain largely undetermined^1,3–8^.

Prior comparisons of genomes from ectomycorrhizal, orchid and ericoid mycorrhizal fungi, wood decayers and soil decomposers have elucidated the mechanisms of several transitions from saprotrophy to mutualism in Dikarya^9–16^. These analyses have shown that multiple lineages of ectomycorrhizal fungi have lost most genes encoding lignocellulose‐degrading enzymes present in their saprotrophic ancestors, explaining the reduced capacity of ectomycorrhizal fungi to acquire C complexed in soil organic matter (SOM) and plant cell walls^17^ and, as a consequence, their increasing dependence on the host plant sugars. The diversity of trophic states in extant ectomycorrhizal fungi may be a consequence of their multiple origins from saprotrophic ancestors with varied decay capabilities, including white and brown rot wood decayers, and soil and litter decomposers^3–5^. However, the extent to which ectomycorrhizal fungi make use of their secreted plant cell wall degrading enzymes (PCWDEs) and microbial cell wall degrading enzymes (MCWDE) to decay or decompose SOM is not well understood^17–24^.

Despite their ecological prominence, much remains to be learned about the evolution and functional diversification of mycorrhizal symbionts and the crucial acquisitions that allow colonization of and nutrient exchange with plants^3,25^. Here, we present a combined analysis of 135 fungal genomes from 73 saprotrophic, endophytic and pathogenic fungal species, and 62 mycorrhizal fungal species, including 29 new mycorrhizal genomes. This study approximately doubles the number of published genomes of mycorrhizal fungi, and it samples major groups, for which there were previously no symbiotic genomes available, including Russulales, Thelephorales, Phallomycetidae, and Cantharellales. These groups are important, because they (1) are often ecologically dominant (Russulales and Thelephorales), (2) represent early diverging clades for which no ectomycorrhizal genomes were previously available (Phallomycetidae and Cantharellales), and (3) include groups that arose before (Cantharellales) or after the origin of ligninolytic peroxidases (class II POD) implicated in white rot^26^.

This dataset presents an opportunity to carry out a broader analysis of the evolution of saprotrophic capabilities than that we previously attempted in our large-scale comparative analysis of Agaricomycetidae^12^. In the present study, we hypothesize that the evolutionary mechanisms in action in Agaricomycetidae can be traced back to the early diverging clades of ectomycorrhizal fungi. To assess gains of ectomycorrhizal lifestyle traits, we discuss the fundamental adaptations that underlie convergent evolution of ectomycorrhizal fungi, including the loss of some metabolic functions, such as PCWDEs, and the acquisition of small secreted effector-like proteins that may facilitate the accommodation of symbiotic fungi within their host plants. We also compare additional symbiosis-related functional traits, such as nitrogen and phosphate acquisition. Finally, we investigate the age distribution of symbiosis-upregulated genes across a phylogenetically representative set of ectomycorrhizal fungi, using a phylostratigraphic approach to resolve lineage-specific and conserved elements of symbiotic transcriptomes. We show that transitions from saprotrophy to ectomycorrhizal symbiosis involve widespread losses of degrading enzymes acting on lignocellulose, co-option of genes present in saprotrophic ancestors to fulfill novel symbiotic functions, diversification of lineage-specific symbiosis-induced genes, proliferation of transposable elements (TEs), and divergent genetic innovations underlying the convergent origins of the ectomycorrhizal guild.

## Results

### Main features of mycorrhizal genomes

**Fig. 1:**
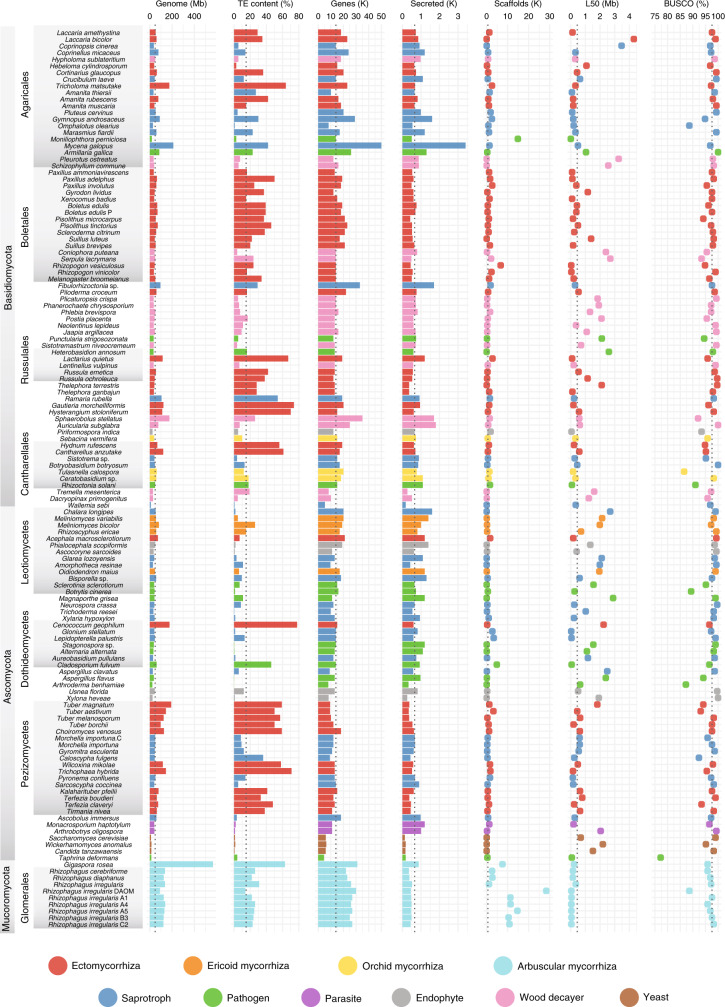
General features of the 135 fungal genomes analyzed. Genome and assembly features. The different lifestyles (e.g., ectomycorrhiza) are color coded, see bottom panel. The dotted lines show median values. Genome (Mb): genome size in Mb, TE content (%): coverage (%) of transposable elements (TE) in assemblies, Genes (K): number of predicted genes, Secreted (K): number of predicted secreted proteins, Scaffolds (K): number of scaffolds, L50 (Mb): N50 length, BUSCO (%): genome completeness in % based on BUSCO survey. Source data Fig. 1.

**Fig. 2:**
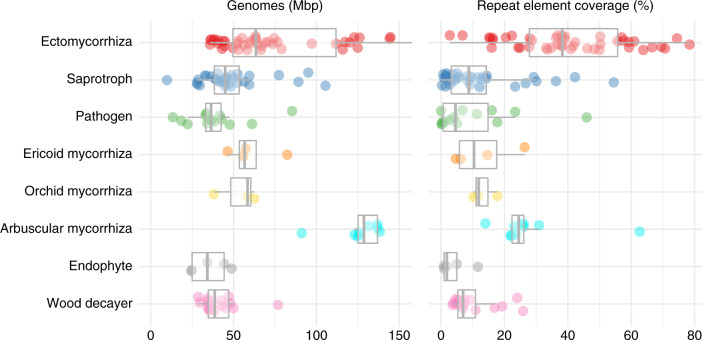
The distribution of genome size (in Mb) and repeat element coverage (%) for each lifestyles. The boxes represent median, upper, and lower quartiles with the whiskers showing minimal and maximal values, and outliers in circle. The small dots show single observations. The number of species per lifestyle is as follows: ectomycorrhizal fungi (*n* = 45), arbuscular mycorrhizal fungi (*n* = 10), ericoid mycorrhizal fungi (*n* = 4), orchid mycorrhizal fungi (*n* = 3), endophytes (*n* = 5), pathogens (*n* = 14), soil/litter saprotrophs (*n* = 32), and wood decayers (*n* = 17). Source data Fig. 1.

We compared 62 draft genomes from mycorrhizal fungi, including 29 newly released genomes, and predicted 9344–31,291 protein-coding genes per species (see “Methods”, Supplementary Information and Supplementary Data 1). This set includes new genomes from the early diverging fungal clades in the Russulales, Thelephorales, Phallomycetidae, and Cantharellales (Basidiomycota), and Helotiales and Pezizales (Ascomycota). We combined these mycorrhizal fungal genomes with 73 fungal genomes from wood decayers, soil/litter saprotrophs, and root endophytes (Fig. 1 and Supplementary Data 2). There was little variation in the completeness of the gene repertoires, based on Benchmarking Universal Single-Copy Orthologs (BUSCO) analysis (coefficient of variation, c.v. = 7.98), despite variation in assembly contiguity (Fig. 1). Genome size varied greatly within each phylum, with genomes of mycorrhizal fungi being larger than those of saprotrophic species (Figs. 1 and 2, and Supplementary Data 2; *P* < 0.05, generalized least squares with the Brownian motion model (GLS)). Glomeromycotina had exceptionally large genomes (Figs. 1 and 2, and Supplementary Data 2), with *Gigaspora rosea* having the largest genome (567 Mb) among the 135 fungi compared^27^.

### TEs in mycorrhizal genomes

**Fig. 3:**
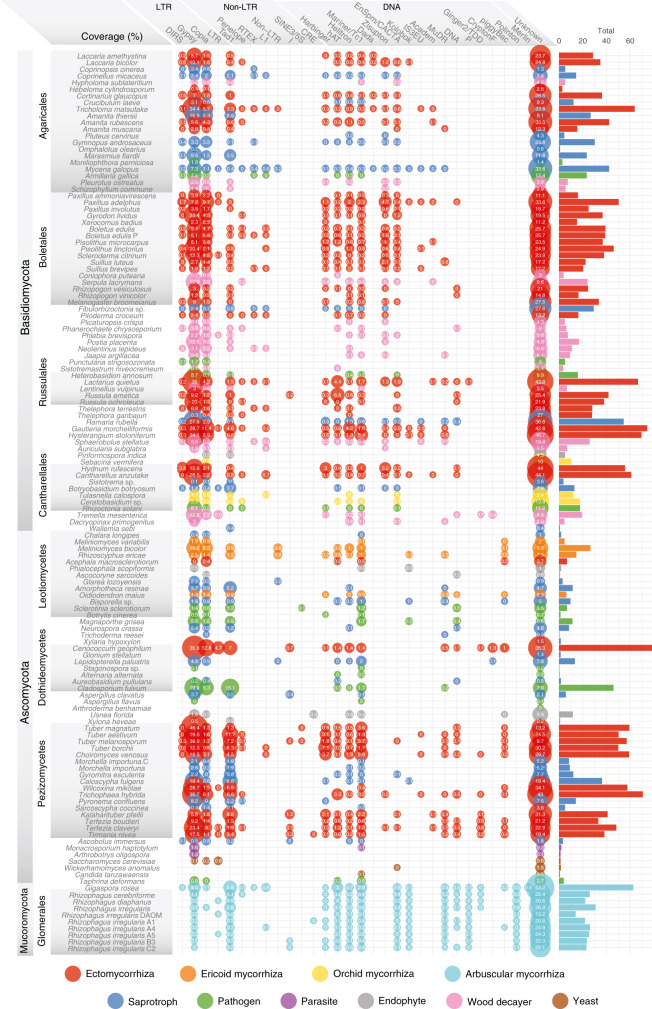
Distribution and coverage (%) of transposable elements families in genome assemblies. The bubble size is proportional to the coverage of each of TE family (% indicated inside bubbles). The bars show the total coverage per genome. See also Supplementary Data 2–4. Source data are provided as a Source data Fig. 1.

The main driver of genome inflation appeared to be repeat content (Fig. 3 and Supplementary Data 3; *P* < 0.05 GLS), such as long terminal repeat retrotransposons (LTRs), which ranged from 0.01 to 46.4% of the assembly (Fig. 3 and Supplementary Data 3). The distribution of TE categories varies between ectomycorrhizal fungal taxa even within the same fungal orders or genera (Fig. 3), indicating that invasions by different TE families took place independently in different clades. However, the total TE coverage in genomes of ectomycorrhizal Ascomycota and Glomeromycota was significantly higher than in Basidiomycota (*P* < 0.05, GLS; Supplementary Data 3). In Ascomycota, the most abundant TE families are LTRs, such as *Gypsy* and *Copia*, and non-LTR I, whereas in Basidiomycota, *Gypsy* and *Copia* LTRs, *Tad1*, helitrons, and Zizuptons are abundant (Fig. 3). Lifestyle has a higher impact than phylogeny on TE coverage with a significantly higher TE content in ectomycorrhizal symbionts compared to other lifestyles for several TE categories (14–35% of total variances, *P* value <0.05; PERMANOVA) (Supplementary Fig. 1a, b, and Supplementary Data 3 and 4). In Agaricales, a large proportion of LTR insertions occurred during the past 5 million years (Mya) in *Amanita rubescens*, *Laccaria bicolor*, *Laccaria amethystina*, and *Cortinarius glaucopus*, while the major bursts are more ancient in *Tricholoma matsutake* (Supplementary Fig. 1c). These LTRs have been proliferating over the past 5–6 Mya for *Tuber melanosporum*, * Tuber aestivum*, and *Choiromyces venosus*, while LTR accumulation in *Tuber magnatum* occurred between 6 and 14 Mya (ref. ^16^).

### Phylogeny, orthologous, and paralogous genes

**Fig. 4:**
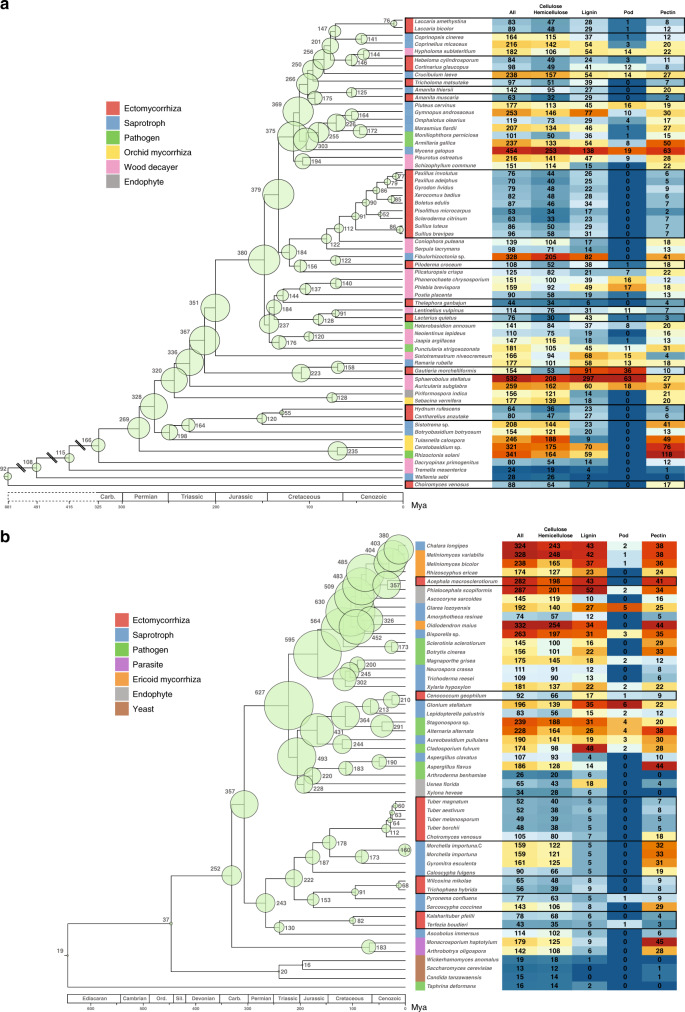
Evolution of gene families encoding PCWDEs in Basidiomycota and Ascomycota. **a** Basidiomycota. **b** Ascomycota. Fungal taxa are displayed according to their phylogeny (left panel). Bubbles with numbers at the tree nodes represent the total number of genes coding for total PCWDEs (intracellular and secreted) for ancestral nodes determined by COMPARE. The bubble size is proportional to the number of PCWDE genes. Heat maps contain the number of genes coding for substrate-specific PCWDEs for the extant species. Relative abundance of genes is represented by a color scale, from the minimum (blue) to maximum (red) number of copies per species. Ectomycorrhizal fungi are framed with a black line. See also Supplementary Data 8 and Supplementary Fig. 5. The tree is a chronogram estimated with r8s on the basis of a maximum likelihood phylogeny inferred with RAxML. The geological timescale (in million year) is indicated at the bottom. Source data are provided as a Source data Fig. 2.

Reconstructed phylogenetic relationships and estimated divergence dates are generally consistent with the dates recovered by previous studies. For example, we estimated the age of the most recent common ancestor (MRCA) of the Agaricomycetidae to be 132 Mya (Fig. 4a), while it was estimated to be 149 or 125 Mya in phylogenomic analyses by Floudas et al.^26^ and Kohler et al.^12^, but 191–176 Mya in a multigene megaphylogeny by Varga et al.^28^. We estimated the ages of the MRCAs of Agaricomycetes, Agaricales, Polyporales, Russulales, and Boletales to be 280, 116, 104, 88, and 82 Mya (Fig. 4a), respectively. We estimated the MRCA of Pezizomycotina at 326 Mya (Fig. 4b), while Floudas et al.^26^ obtained a mean age of 344 Mya.

We inferred gene families from the predicted proteomes using gene family clustering. A total of 68,923 and 129,258 gene families were identified in Ascomycota and Basidiomycota, respectively, and were used to infer orthology and paralogy (Supplementary Data 5). The species in our datasets contained substantial novelty in gene content. Species-specific genes ranged from 994 in *T. melanosporum* to 39,410 in *Mycena galopus*, for a total of 251,392 and 606,378 taxon-specific genes in Ascomycota and Basidiomycota, respectively.The large number of species-specific genes in *M. galopus* is the result of a series of striking expansion of gene families.

### Functional gene categories encoded by mycorrhizal genomes

Hierarchical clustering of the presence and abundance of different Pfam protein domains identified genome-wide patterns of functional domain content among some of these fungi (Supplementary Fig. 2a). Arbuscular mycorrhizal fungi clustered together with Pfam categories showing a substantial differential abundance in genes encoding proteins with NUDIX, tetratricopeptide repeat, BTB/POZ, Sel1 repeat, ubiquitin, and high-mobility group box domains, corroborating our previous study^27^. This comparison also emphasizes some of the unique aspects of the genomes from the ectomycorrhizal ascomycete *Acephala macrosclerotiorum* (Helotiaceae) and ericoid mycorrhizal fungi (e.g., *Oidiodendron maius*, *Meliniomyces* species), such as the expansion of genes encoding proteins with FAD and AMP-binding domains, aldehyde dehydrogenases and sugar transporters. They share their Pfam pattern with soil saprotrophic ascomycete species, such as *Chalara longipes* and the dark septate endophyte (DSE) *Phialocephala scopiformis*. Ectomycorrhizal fungi in Basidiomycota are grouped with soil saprotrophs and wood decayers, and displayed no specific pattern in their primary and secondary metabolism gene repertoires that may explain their symbiosis-related ability. Similarly, hierarchical clustering of the presence and abundance of the different membrane transporters and transcriptional regulators revealed no specific pattern(s) for ectomycorrhizal fungi (Supplementary Fig. 2b, c).

### Predicted secretomes of saprotrophs and symbiotrophs

As secreted proteins play a key role in SOM decomposition and symbiosis development, we compared predicted secretomes, including carbohydrate-active enzymes (CAZymes), lipases, proteases, and other secreted proteins, such as effector-like small secreted proteins (SSPs; Supplementary Data 6). The number of genes encoding secreted proteins represents 4.6–5.7% of the total protein repertoire for Basidiomycota and Ascomycota, respectively (Supplementary Fig. 3). The proportions of secreted proteins within the different protein categories were consistent in both phyla (Supplementary Fig. 3, and Supplementary Data 6 and 7). About half of the secreted proteins were SSPs (Supplementary Fig. 3a and Supplementary Data 7c). Only secreted CAZymes and SSPs showed significant differences in relative abundance among lifestyles (*P* < 0.01; generalized Campbell and Skillings procedure; Supplementary Fig. 3 and Supplementary Data 8), with orchid and ericoid mycorrhizal symbionts possessing the largest CAZyme repertoires. On the other hand, the average number of secreted proteases and lipases is similar in saprotrophs and symbiotrophs. No expansion of gene families coding for secreted proteases, secreted phosphatases, and phytases were found in ectomycorrhizal fungi (Supplementary Fig. 3c).

### Losses of PCWDEs

**Fig. 5:**
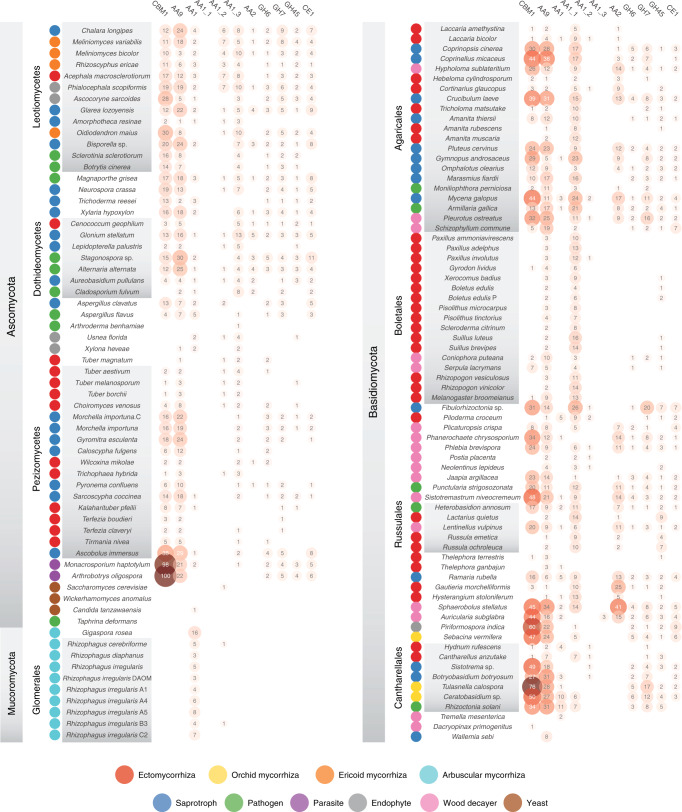
Distribution of key secreted PCWDEs in analyzed fungi. Bubbles with numbers contain the number of genes coding for a series of secreted PCWDEs needed for cellulose and lignin degradation. Taxa are color coded according to their lifestyle (see bottom panel). See also Supplementary Data 6a, j. Left panel, Ascomycota and Mucoromycota; right panel: Basidiomycota. Source data are provided as a Source data Fig. 3.

In Basidiomycota, ectomycorrhizal species contain significantly fewer secreted CAZymes acting on cellulose, hemicellulose, pectins, lignin, suberins, and tannins (*P* < 0.01, the generalized Campbell and Skillings procedure; Fig. 5, Supplementary Fig. 3c and Supplementary Data 6) than all other ecological guilds, i.e., lifestyles. They are also reduced in secreted auxiliary activity (AA) enzymes (including class II PODs, laccases, lytic polysaccharide monooxygenases (LPMO)), carbohydrate esterases (CE), polysaccharide lyases (PL), and associated cellulose-binding modules (e.g., CBM1; Supplementary Figs. 3–5, and Supplementary Data 7 and 8). Notably, there is no invertase GH32 gene in their genome, except for *Cantharellus anzutake*, implying that ectomycorrhizal basidiomycetes are unable to use sucrose directly from the plant. Except for *Acephala macrosclerotiorum*, the number of PCWDEs in ectomycorrhizal Ascomycota is lower than those of most of other groups, excluding yeasts and arbuscular mycorrhizal fungi (Figs. 4 and 5, and Supplementary Figs. 3–5). According to RedoxiBase (http://peroxibase.toulouse.inra.fr), none of the class II PODs of ectomycorrhizal species are ligninolytic (i.e., ligninolytic POD or LiP), except those of *Gautieria morchelliformis* (see below; Supplementary Fig. 4 and Supplementary Data 6p). Below, we discuss the PCWDEs of ectomycorrhizal fungi, emphasizing newly sampled lineages with previously unknown decomposition capacity.

Cantharellales arose before the origin of ligninolytic class II POD^26,28–30^. Both saprotrophs (*Botryobasidium botryosum*, *Sistotrema* sp.) and orchid symbionts (*Tulasnella calospora*, *Ceratobasidium* sp.) in Cantharellales possess large sets of enzymes acting on cellulose, hemicellulose, and pectins, including LPMOs (AA9; Figs. 4 and 5, and Supplementary Figs. 3–5). We sequenced the first genomes of ectomycorrhizal Cantharellales, *Hydnum rufescens*, and *C. anzutake*, and found that they have highly reduced repertoires of secreted PCWDEs. In this regard, the ectomycorrhizal symbionts of Cantharellales are more similar to ectomycorrhizal Agaricales and Boletales than to orchid symbionts of Cantharellales. These results provide another independent example of convergent loss of PCWDEs in ectomycorrhizal lineages, and highlight the different decomposition capacities of two guilds of plant symbionts.

In contrast to Cantharellales, Phallomycetidae diverged shortly after the evolution of ligninolytic class II POD. The one previously published genome from this group, the saprotrophic “cannonball fungus”, *Sphaerobolus stellatus*, has an astonishingly large repertoire of PCWDEs, including 63 class II PODs. The two genomes of ectomycorrhizal Phallomycetidae reported here, *G. morchelliformis* (Gomphales) and *Hysterangium stoloniferum* (Hysterangiales)^31^, have diverse PCWDEs acting on cellulose, hemicellulose, pectins, and lignin, with 31 and five genes encoding ligninolytic class II PODs, respectively (Figs. 4 and 5, Supplementary Figs. 3–5 and Supplementary Data 8). Although many *Ramaria* species (Gomphales) form ectomycorrhiza, the newly sequenced *Ramaria rubella* (subgenus *Lentoramaria*) is likely a litter decomposer, which is consistent with its possession of 13 class II PODs, 6 cellulose-acting LPMOs, and GH6 and GH7 cellobiohydrolases. Thus, a robust suite of PCWDEs appears to be a characteristic of both saprotrophs and ectomycorrhizal species in Phallomycetidae.

Ectomycorrhizal Russulales and Thelephorales, for which we report the first genomes, both have highly reduced complements of PCWDEs. The two Thelephorales species (*Thelephora terrestris* and *T. ganbajun*) resemble Boletales in having a highly reduced suite of the major enzymes acting on crystalline cellulose and lignin (three cellulose-acting LPMOs, no class II POD, and no GH6 or GH7). Ectomycorrhizal Russulales (*Lactarius quietus, Russula emetica*, and *Russula ochroleuca*) also have small repertoires of PCWDEs (no CBM1, one or two cellulose-acting LPMOs, no GH6 or GH7, no CE1, and no LiP), but they retain one atypical Mn POD gene (Supplementary Data 6p). In contrast, saprotrophic Russulales (e.g., *Lentinellus vulpinus*) have a typical white rot array of PCWDEs.

In Ascomycota, species in Tuberaceae followed the general symbiotroph trend, except *C. venosus*, which has a large complement of PCWDEs (Figs. 4a and 5, see ref. ^16^). Other ectomycorrhizal Pezizales, such as the newly sequenced *Wilcoxina mikolae* and *Trichophaea hybrida* (Pyronemataceae), also have few PCWDEs. The desert truffles *Kalaharituber pfeilii* and *Terfezia boudieri* (Pezizaceae) both have ectomycorrhiza-like restricted suites of PCWDEs, but they differ in the numbers of genes encoding cellulose-acting LPMOs (ten and one copies, respectively) and they encode one copy of GH32 invertase. Like other ectomycorrhizal species*, A. macrosclerotiorum* contained no class II PODs, but had a large set of PCWDEs (282 genes, including 17 cellulose-acting LPMOs, and genes encoding GH6, GH7, GH32, GH45, PL1, and PL3; Figs. 4 and 5, Supplementary Figs. 3–5 and Supplementary Data 8). Closely related taxa to *A. macrosclerotiorum* mainly contain DSEs, such as *P. scopiformis*, saprotrophs, and pathogens, which are characterized by a larger set of PCWDEs compared to ectomycorrhizal fungi. To assess whether this large repertoire of PCWDEs is expressed during symbiosis development, we carried out transcript profiling of *Pinus sylvestris*–*A. macrosclerotiorum* ectomycorrhizas using RNA-Seq. The fungal symbiont develops a dense Hartig net within the root cortex, but no mantle sheath (Supplementary Fig. 6a). Transcript profiling of ectomycorrhizas showed that most of the PCWDEs are not induced in symbiotic tissues (Supplementary Fig. 6b and Supplementary Data 16), suggesting a tight transcriptional control of the PCWDE gene expression.

### MCWDEs are retained in ectomycorrhizal fungi

We explored the genetic capabilities of the sequenced fungi to decompose microbial (i.e., bacterial and fungal) cell walls by comparing PCWDE to MCWDE repertoires (Figs. 4 and 5, Supplementary Figs. 3–5 and Supplementary Data 7). The proportion of MCWDE (acting on chitin, glucans, mannans, and peptidoglycans) in ectomycorrhizal fungi is similar to that in saprotrophs. Thus, ectomycorrhizal fungi have generally retained MCWDE genes (e.g., chitinases, ß-1,3-glucanases) although they have lost most PCWDEs (e.g., endo- and exocellulases). Ectomycorrhizal fungi may use secreted MCWDE to scavenge nitrogen compounds trapped in SOM (e.g., chitin) by selectively using these hydrolytic enzymes in addition to oxidative mechanisms^32^. Some of these chitin-, glucan-, and mannan-active enzymes are likely involved in fungal cell wall remodeling during complex developmental processes, such as ectomycorrhiza and sporocarp formation^10,11,33^.

### Mycorrhizal development is driven by gene co-option

**Fig. 6:**
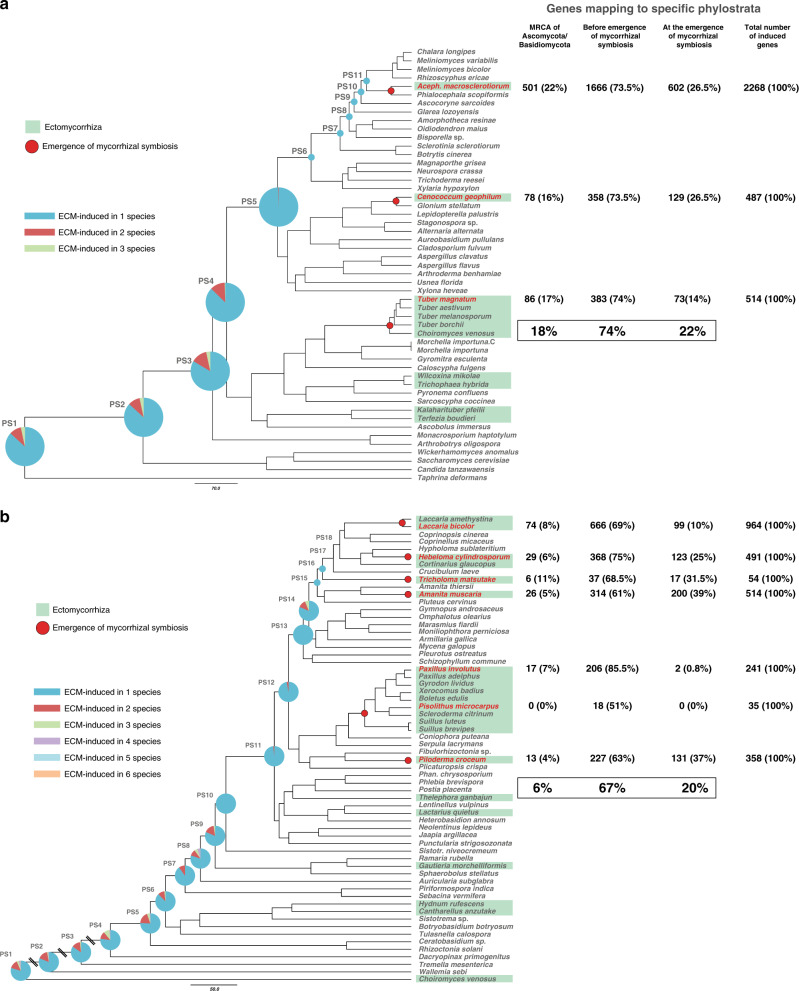
Phylostratigraphy for ectomycorrhiza-specific upregulated genes. Pie charts on the time-calibrated trees represent the number of gene clusters containing ectomycorrhiza-specific upregulated genes from the species compared. Numbers and percentage of genes mapping to phylostrata are shown on the right of the trees. Ectomycorrhizal species used for the comparison are in green boxes. Upregulated genes were selected according to FDR adjusted *P* value < 0.05. **a** Ascomycota, the fold change (FC) used for defining ectomycorrhiza-specific upregulated genes was FC ≥ 5. **b** Basidiomycota, the FC used for defining ectomycorrhiza-specific upregulated genes was FC ≥ 5. See Supplementary Data 9 for the identified phylostrata containing ectomycorrhiza-upregulated genes and enrichment statistics. Source data are provided as a Source data Fig. 4.

We used a phylostratigraphic approach to characterize the evolutionary origins of ectomycorrhizal lineages on the basis of gene functions in extant organisms. We examined the age distribution of genes induced at different stages of ectomycorrhiza establishment, so-called symbiosis-induced genes, by defining phylogenetic ages (phylostrata), that correspond to internal nodes of the tree along the lineage leading from the root to the symbiotic species for which transcriptomic data are available (Fig. 6, Supplementary Fig. 7 and Supplementary Data 9). In Ascomycota and Basidiomycota, an average of 74% and 67% of the ectomycorrhiza-induced genes predated the evolution of ectomycorrhizal symbiosis, respectively (Fig. 6). Approximately 6% and 18% of these genes were already present in the MRCAs of the Ascomycota and that of the Basidiomycota (e.g., for *L. bicolor*, hydrophobins, GH131, GH28, and CBM1_GH5), respectively (Fig. 6 and Supplementary Fig. 7). No specific phylostrata was enriched in symbiosis-induced genes (Fisher’s exact test, *P* < 0.005; Supplementary Data 9b), suggesting that there was no distinguished period during ectomycorrhiza evolution caracterized by an excess number of gene birth events. These observations imply that symbiosis-induced genes have mostly been co-opted for ectomycorrhiza development during evolution from saprotrophic ancestors. However, the phylostratigraphic analysis also suggested that an average of 9–22 % (in Ascomycota; Fig. 6a) and 19–20 % (in Basidiomycota; Fig. 6b) of ectomycorrhiza-induced genes are restricted to specific mycorrhizal lineages. Given our current taxon sampling, it is difficult to distinguish genes that coincidentally evolved with mycorrhiza formation from species-specific orphan genes, except in the case of three clades that comprise more than a single ectomycorrhizal species, Boletales, the genus *Laccaria* and Tuberaceae, which comprise more than a single ectomycorrhizal species. In these clades, the proportion of symbiosis-induced genes that map to the origin of ectomycorrhiza showed a large variation, from 0 to 0.8% in the Boletales (two species), to 10% in *Laccaria*, and 14% in the Tuberaceae (Fig. 6).

**Fig. 7:**
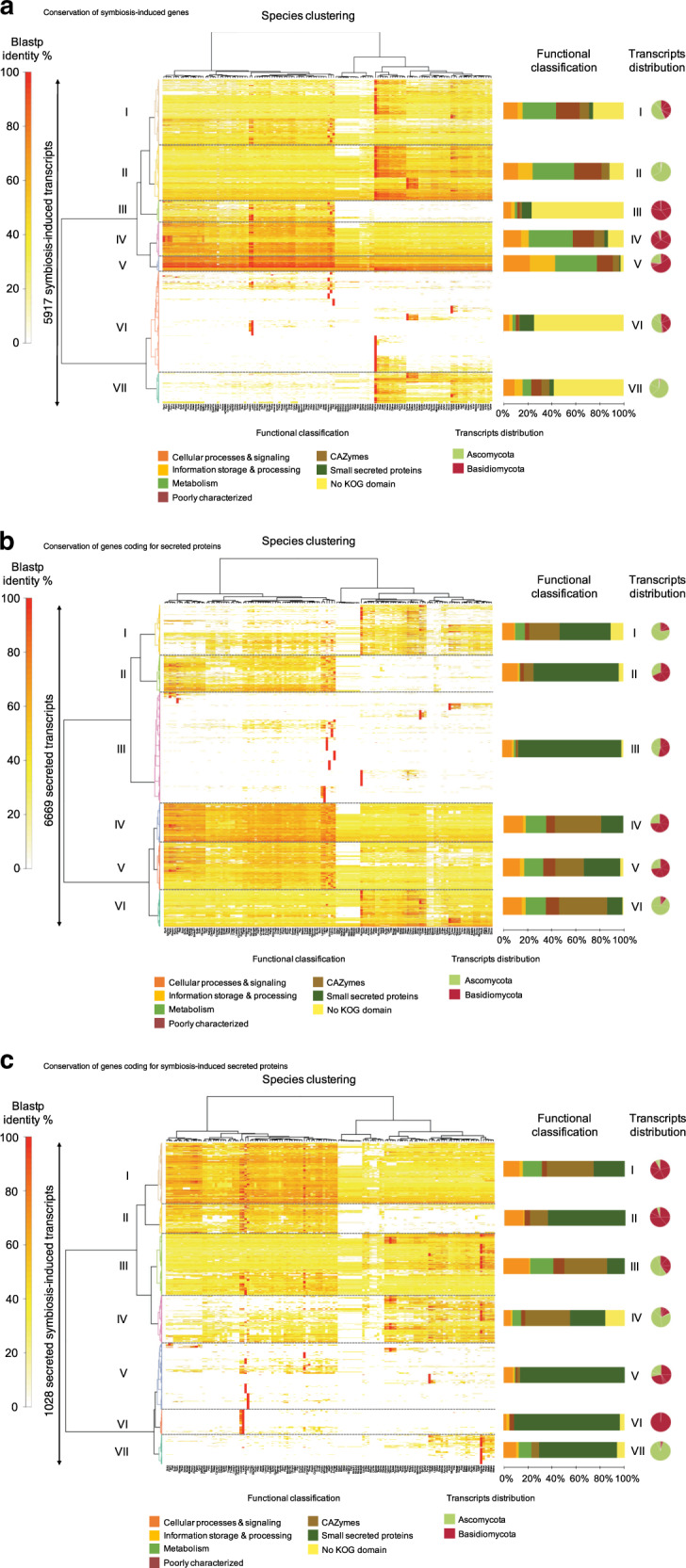
Phylogenetic conservation of ectomycorrhiza-induced genes. We analyzed genes coding for ectomycorrhiza-induced proteins, secreted proteins and ectomycorrhiza-induced secreted proteins among 135 fungi. Heat maps show BLASTP sequence similarity of 5917 ectomycorrhiza-induced proteins (**a**), 6669 genes coding for secreted proteins (**b**), and 1028 ectomycorrhiza-induced  secreted proteins (**c**) from ten ectomycorrhizal fungi, with available symbiotic transcriptomes among 135 genomes of Basidiomycota and Ascomycota. The heat maps depict a double-hierarchical clustering of protein sequences encoded by symbiosis-upregulated genes, genes coding for secreted proteins and genes coding for symbiosis-upregulated secreted proteins (rows, fold change ≥ 5 in symbiotic tissues compared to free-living mycelium, false discovery rate-corrected *P* ≤ 0.05); right panel, functional categories (KOG) are given for each cluster of sequences in % as bargrams. Clusters I–VIII correspond to group of sequences sharing the same level of protein sequence similarity based on BlastP. The distribution of transcripts belonging to Ascomycota and Basidiomycota in each cluster is shown as pie charts. Data were visualized and clustered using R (package HeatPlus^97^). The hierarchical clustering was done by using a Euclidian distance and Ward clustering method. Color scale on the left (white to red) shows the % of sequence identity according to BLASTP. The symbiosis-induced genes were retrieved from the ectomycorrhizal transcriptomes of ten species: *A. macrosclerotiorum* EW76-UTF0540, *A. muscaria* Koide, *C. geophilum* 1.58, *H. cylindrosporum* h7, *L. bicolor* S238N, *P. involutus* ATCC 200175, *P. croceum* F1598, *P. microcarpus* 441, *T. matsutake* 945, and *T. magnatum* (see Supplementary Data 10, 13 and 14). GEO accession codes are provided in the “Methods/Phylostratigraphy” section. Source data are provided as a Source data Fig. 5.

To test our phylostratigraphic results, we assessed the evolutionary conservation of 5917 ectomycorrhiza-induced transcripts identified in ten different ectomycorrhizal interactions among the 135 studied fungal genomes (Fig. 7a). We found that 15.2 % of the 5917 symbiosis-induced genes are shared by all species in our dataset (clusters IV and V). Most encode proteins involved in core metabolic or signaling functions. Genes from cluster III are conserved within Basidiomycota only, while genes from cluster VII are shared by Ascomycota only. Most of them have no known function (i.e., no KOG domain). Altogether, 31.2% of these symbiosis-induced genes are species specific (cluster VI). Most of these genes encode proteins with unknown KOG functions or mycorrhiza-induced small secreted proteins (MiSSPs). The proportion of species-specific, ectomycorrhiza-induced genes is therefore substantial, as reported earlier^10,12,16^ and suggested by our phylostratigraphic analysis.

Next, we examined whether the same or different gene families were co-opted in independent ectomycorrhizal lineages, i.e., whether co-option was convergent or divergent during fungal evolution. We quantified convergence by the extent of overlap among conserved ectomycorrhiza-induced gene families within independent lineages. Conserved ectomycorrhiza-induced genes showed little or no overlap among the analyzed species (Fig. 6 and Supplementary Data 9). For example, there were only eight clusters of ectomycorrhiza-induced genes common to at least four Basidiomycota symbionts (Supplementary Data 9). Similarly, only 12 gene clusters were shared by at least three Ascomycota symbionts (Supplementary Data 9). This low overlap between co-opted genes suggests that independently evolved ectomycorrhizal lineages recruited different ancestral gene families for symbiosis, in addition to a likewise unique set of novel genes, that evolved after the origins of symbiosis^12^.

### Symbiosis-induced secreted proteins in symbionts

The expression of genes encoding secreted and symbiosis-induced secreted proteins from ectomycorrhizal fungi was measured by RNA-Seq profiling in ten ectomycorrhizal associations (Supplementary Methods). By using a BLASTP-based analysis, we assessed the evolutionary conservation of the expressed 6669 secreted proteins (Fig. 7b) and 1028 symbiosis-induced secreted proteins (Fig. 7c) among 135 fungal species (Supplementary Datas 12, 13 and 14). A substantial proportion of the secreted proteins are species-specific SSPs (cluster III, Fig. 7b). In addition, we found that 38.1% of symbiosis-induced secreted proteins are shared by all species of fungi (clusters I and III). Most code for core metabolic or signaling functions and CAZymes. Genes from cluster II (9.3%) are conserved within Basidiomycota only. Genes from cluster IV (21.4%) are conserved mainly in Ascomycota, showed a lower similarity in Basidiomycota and are poorly conserved in Glomeromycota. They encode MiSSPs, CAZymes, proteins with unknown KOG function and proteins of signaling and metabolic pathways. Altogether, 31.2% of these 1028 symbiosis-induced genes are mostly species specific (clusters V–VII). Most of these genes encode MiSSPs, proteins with unknown KOG functions and proteins of signaling pathways. The phylostratigraphic analysis of secreted proteins and MiSSPs corroborated these results (Supplementary Fig. 7).

## Discussion

After the origin of Pinaceae (ca. 200 Mya), ectomycorrhizal symbiosis arose repeatedly, perhaps 80 times or more, across multiple lineages of Mucoromycotina (Endogonales), Ascomycota, and especially Basidiomycota^2–4,8,34,35^. The ancestors of ectomycorrhizal fungi are genetically and ecologically diverse, making this a superb example of convergent evolution^3,7,9,12^. To assess the general shared properties of the lifestyle evolution and functional biology of ectomycorrhizal symbioses, we conducted a comparative analysis of 62 mycorrhizal and 73 non-mycorrhizal fungal species. Our dataset, the most comprehensive so far, includes several major fungal clades that have not been sampled previously for ectomycorrhizal genomes, i.e., Cantharellales, Phallomycetidae, Thelephorales, and Russulales. For the sake of comparison, it also includes genomes of arbuscular mycorrhizal fungi, orchid mycorrhizal fungi, and ericoid mycorrhizal fungi providing a unique opportunity to highlight differences and similarities between the major types of mycorrhizal symbioses.

Our analyses of early diverging clades of ectomycorrhizal fungi (e.g., Cantharellales) support the general view that transitions from saprotrophy to ectomycorrhizal symbiosis involve (1) widespread losses of PCWDEs acting on lignin, cellulose, hemicellulose, pectins, suberins, and tannins, (2) co-option of metabolic and signaling genes present in saprotrophic ancestors to fulfill new symbiotic functions, (3) diversification of novel, lineage-specific symbiosis-induced orphan genes, and (4) massive proliferation of TEs. In addition, they corroborated and extended our previous analyses of the genomes of arbuscular mycorrhizal fungi^27^ and ericoid mycorrhizal fungi^15^. Despite the general trend toward losses of PCWDEs in ectomycorrhizal lineages in both Ascomycota and Basidiomycota, there is considerable diversity in the apparent decay capacities of ectomycorrhizal fungi. For example, in Agaricales (Agaricomycetidae), *C. glaucopus* possesses 12 recently duplicated copies of atypical class II POD genes, which may confer some ability to obtain nutrients from soil phenolic compounds, as previously suggested^36^. However, secreted PODs can play a variety of additional roles, such as biosynthesis of cell wall melanins, detoxifying the immediate hyphae environment, or/and converting plant polymers into more oxidized, recalcitrant components of SOM. Similarly, the genome of *T. matsutake* (also Agaricales) encodes two GH7 cellobiohydrolases, in agreement with its known facultative saprotrophic ability^37^. Ectomycorrhizal fungi that evolved from brown rot fungi in the Boletales (also Agaricomycetidae), such as *Paxillus involutus*, appear to have adapted the oxidative decomposition system from their saprotrophic ancestors to liberate N entrapped in decaying SOM^38–41^. Their secreted proteases, LPMOs, and laccases may act in concert to decay available SOM compounds. Alternative hypotheses for the role of the remaining PCWDEs in ectomycorrhizal fungi include the remodeling of root cell walls during host colonization^42^.

Among the newly sampled fungal groups, ectomycorrhizal Phallomycetidae and Cantharellales present highly variable suites of PCWDEs. Most striking is the ectomycorrhizal *G. morchelliformis* (Gomphales, Phallomycetidae), which has 25 class II manganese PODs (MnP) that may be involved in the decay of SOM and/or detoxification of soil polyphenolic compounds, as well as numerous enzymes active on cellulose and hemicellulose. The class II PODs of *G. morchelliformis* are members of a gene family that underwent expansion after the divergence of Auriculariales and other Agaricomycetes. In contrast, Cantharellales evolved prior to the diversification of class II LiP^30^, and none of its species—saprotrophic or mycorrhizal—possesses these ligninolytic enzymes. In contrast to their saprotrophic cousin *Sistotrema* sp., *C. anzutake* and *H. rufescens* are ectomycorrhizal species of Cantharellales that lack not only class II LiP, but also many glycoside hydrolases acting on cellulose, hemicellulose and pectins, LPMOs, and CBM1 motifs that are needed for plant cell wall and SOM decomposition. In this regard, they resemble some of the most derived ectomycorrhizal Agaricomycetidae with highly reduced saprotrophic capabilities, such as species of Boletales. The ectomycorrhizal Russulales and Thelephorales that we sampled also conform to this model of a greatly reduced saprotrophic apparatus in prominent symbiotic fungi playing major roles in forest ecosystems.

Ectomycorrhizal fungi are not all depauperate in PCWDEs. Species such as *A. macrosclerotium* in the Leotiomycetes, may represent transitional steps from pure saprotrophy toward pure ectomycorrhizal symbiosis. Such taxa could be capable of facultative saprotrophy, i.e., SOM decomposition. As suggested for ericoid mycorrhizal fungi, which exhibit a large PCWDE repertoire^15^, a dual saprotrophic/mutualistic habit may provide greater ecological flexibility and fitness under specific environmental conditions. Moreover, in such lineages, evolutionary reversal to saprotrophy might be possible (although that has not been demonstrated). We showed that the PCWDEs of *A. macrosclerotium* are downregulated at the transcriptional level during the symbiotic interaction to avoid triggering plant defense reactions and/or digesting host root tissues. This suggests that loss of genes encoding PCWDEs is a consequence of, but not a requirement for, the evolution of ectomycorrhizal mutualisms. What remains to be determined is why ectomycorrhizal fungi do not retain their ancestral dual lifestyle, and how the ectomycorrhizal associations become so intimate that saprotrophic capabilities are irretrievably lost. The lack of invertase and sucrose transporter genes in most ectomycorrhizal fungi means that they are unable to use apoplastic sucrose released by their host plant. They fully rely on their partner for their glucose supply, a mechanism reinforcing their dependence on the plant partner.

Our results, and those of prior studies^3,14^, suggest that the predominant mechanism for the transition from saprotrophy to ectomycorrhizal symbiosis appears to involve restricted secretion of hydrolytic enzymes acting on plant cell walls (whether via regulatory shifts or gene loss), which enables the symbiont to be accommodated in roots. An example of an ultimate adaptation to symbiosis is illustrated by the arbuscular mycorrhizal fungi that are obligate symbionts. They display the lowest repertoire of PCWDEs of the current set of sequenced mycorrhizal fungi. Unfortunately, no genomic data exist yet to support the origin of arbuscular mycorrhizal fungi from saprotrophic CAZyme-rich Mucoromycotina. In contrast, ericoid mycorrhizal fungi and orchid mycorrhizal fungi have the higher set of PCWDEs supporting their dual saprotrophic/symbiotic lifestyles^3,15^. We thus propose that the PCWDE repertoire is reflective of the age of the symbiosis along the saprotrophy to symbiosis continuum with the arbuscular mycorrhizal symbiosis emerging in the early Devonian (393–419 Mya), the ectomycorrhizal symbiosis during the Jurassic (ca. 200 Mya) and mycorrhizal associations with Ericaceae species to the Cretaceous (ca. 117 Mya)^4^.

Further insight into the differences in gene composition between saprotrophic and symbiotic fungi was obtained by focusing on genes involved in adaptations to the host plant habitat in which symbionts thrive. Novel genes, such as effector-like MiSSPs, are likely required for symbiosis development (e.g., dampening of plant defense reactions^43,44^) and metabolism^45^, and have evolved in every ectomycorrhizal lineage. Transcript profiling of several ectomycorrhizal interactions has shown that 7–38% of genes that are upregulated during symbiosis are species-specific genes, i.e., they are restricted to a single mycorrhizal species. Among all symbiosis-upregulated genes, 8–28% encode candidate secreted effector-like MiSSPs. Their functional analysis is hampered by the the lack of high-throughput genetic engineering techniques for ectomycorrhizal fungi, but some of these MiSSPs have been functionally characterized, including LbMiSSP7, LbMiSSP8, LbMiSSP7.6, and PaMiSSP10 (refs. ^44,46–48^). The evolution of innovations, such as the control of the host plant immunity, development, and metabolism by these lineage-specific symbiotic effectors was likely a necessary step to colonized the new niches represented by tree roots. Some of the effector-like SSPs, but also structural SSPs like hydrophobins, may have evolved from SSPs used by the saprotrophic ancestors to communicate or compete in the soil and wood environments^,46,49^.

Novel and recently evolved genes, including MiSSPs, are undoubtedly responsible for the particular attributes of individual mycorrhizal lineages. Indeed, arrays of ectomycorrhiza-induced genes are unique to each specific clade with between 14 and 39% of symbiosis-upregulated genes being species-specific genes with no known function^12^ (present study). At the same time, our phylostratigraphic analysis showed that a large proportion of genes that are upregulated in ectomycorrhizas arose long before the evolution of the mutualistic associations. In other words, numerous genes used by saprotrophic ancestors were co-opted for the symbiotic lifestyle. Important ecological traits, such as N and P acquisition traits (e.g., organic N- and P-degrading secreted enzymes) were already present in free-living saprotrophic ancestors of ectomycorrhizal symbionts. Despite the well-documented ability of ectomycorrhizal fungi to hydrolyze organic phosphate compounds and scavenge nitrogen through the degradation of litter proteins accumulating in soil litter, we found that ectomycorrhizal genomes generally contain a similar number or less copies of genes coding for secreted N- and P-targeting hydrolases than saprotrophs, pathogens, or ericoid mycorrhizal fungi.

One of the most striking genomic features of mycorrhizal fungi is their high content in TEs. A large proportion of the genomes of mycorrhizal fungi can be ascribed to past transposition, providing a major contribution to their genome landscape. It has been proposed that the lack of sexual recombination (i.e., asexual reproduction) can favor the uncontrolled proliferation of TEs^47^. It may be true for arbuscular mycorrhizal fungi with no known sexual reproduction, but this hypothesis cannot stand for the ectomycorrhizal fungi investigated in the present study as they are known to produce ascocarps or basidiocarps. In biotrophic pathogens, such as *Blumeria graminis* f. sp. *hordei* and *B. graminis* f. sp. *tritici*, it has been proposed that burst(s) of TEs offer a template for rapid evolution of virulence genes by duplications, small-scale rearrangements, or deletions^48^. It is tempting to speculate that the observed TE proliferation(s) play a key role in promoting the rapid evolution of symbiosis-related factors, such as MiSSPs.

Collectively, our results suggest that each independently evolved ectomycorrhizal lineage uses species-specific novel genes, as well as members of ancestral gene families to develop symbiotic interactions, indicating that divergent genetic innovations underlie the convergent origins of the ectomycorrhizal guild. Thus, the functional heterogeneity in symbiosis-related gene networks in ectomycorrhizal fungi is not only found among species-specific genes as evidenced for MiSSPs^3,14,43–45,50,51^, but also in conserved genes (e.g., hydrophobins) that predate the gains of mycorrhizal symbiosis.

The large number of independently evolved ectomycorrhizal lineages raises questions as to why the emergence of this guild is so common in nature. Furthermore, the absence of known reversals to the saprotrophic lifestyle suggests that genetic traits underlying the transitions to ectomycorrhizal lifestyles represent fixed evolutionary transitions. Future studies should focus on conditions that may predispose some groups to evolve ectomycorrhizal symbioses, or other mechanisms that increase the likelihood of convergent evolution^52–55^. The present study roughly doubles the number of published ectomycorrhizal genomes. Even greater sampling of key lineages, particularly early divergent  clades, is needed to elucidate the macro- and microevolutionary mechanisms that were responsible for the diversification of ectomycorrhizal fungi.

## Methods

### Strains and fungal material used for genome sequencing

Fungal strains used for genome sequencing are described in Supplementary Data 1. Assignment of individual taxa to functional guilds was based on common categorizations in the literature^34,56,57^. DNA was extracted with a modified cetyltrimethylammonium bromide protocol^12^.

### Genome sequencing, assembly, and annotation

All sequencing, assembly, and annotation was performed at JGI. Genome sequencing was done with either Illumina or Pacific Biosciences (PacBio) technology, or both (“hybrid”). Genomes were assembled from Illumina reads using ALLPATHS-LG^58^, followed by patching using PacBio with PBJelly^59^ in the case of hybrid assemblies. All other genomes were assembled from PacBio reads using Falcon^60^ or Celera Assembler^61^. Mitochondrial genomes were assembled separately. All transcriptome sequencing was done with Illumina only, and subsequently assembled into putative transcripts using either Rnnotator^11^ or Trinity^62^. Each genome was annotated using the JGI Annotation Pipeline^63,64^, aided by the transcriptome when available. Detailed methods are described in the Supplementary Methods.

### Organismal phylogeny

We assembled two datasets, one of Ascomycota that included 51 genomes and one of Basidiomycota that included 62 genomes. We opted for analyzing Ascomycota and Basidiomycota genomes separately, and only fungal genomes to improve computational efficiency and because the origins of the ectomycorrhizal symbioses are shallow evolutionary events, so we reasoned that additional resolution cannot be added by including non-fungal outgroup taxa. The computational step of similarity-based clustering, multiple sequence alignments, and tree inference are particularly sensitive to the phylogenetic breadth that the species cover. Analyzing Ascomycota and Basidiomycota separately improved the accuracy of our predictions, by not having to analyze as deep nodes of the tree as the Dikarya (MRCA of Ascomycota and Basidiomycota). We performed all-vs-all Blast with mpiBlast 1.6.0 (ref. ^65^) and clustered proteins into gene families by Hifix 1.0.5 (ref. ^66^), using default parameters. Clusters having 0.5*n*–2*n* proteins (*n* = number of species) were aligned by PRANK140603 (ref. ^67^) with default settings, and trimmed with trim-Al 1.4.rev15 (ref. ^68^) to remove ambiguously aligned regions (with the argument -gt 0.1). Maximum likelihood (ML) gene trees were inferred using FastTree 2.1.10 (refs. ^69,70^) under the Whelan and Goldman (WAG) model of protein evolution with gamma-distributed rate heterogeneity. We excluded gene trees with deep paralogs to identify single-copy gene. Gene trees of not strictly single-copy gene families were checked if the duplications are deep in the tree, or species specific. In those cases where the duplications were species specific, the gene was marked as suitable for phylogenetic inference and the protein that was closest to the root (in terms of patristic distance) for each species was chosen for inclusion in the phylogenetic analyses.

Single-copy genes were realigned using PRANK v. 150803 (ref. ^67^) with the default settings and one round of alignment improvement. Poorly aligned regions were removed using trim-Al v1.2 (ref. ^68^) with the -strict setting. The alignments were concatenated into a supermatrix using a custom Perl script, excluding single alignments that were <50 amino acids long. We included genes in the supermatrix only if they were present in >40 and >50 Ascomycota and Basidiomycota species, respectively. ML phylogenies were inferred using RAxML 8.2.4 (ref. ^71^) with 100 bootstrap replicates and a partitioned model, where each gene was treated as a separate partition. The PROTGAMMAWAG model was used for each partition. The resulting phylogenies were used in the molecular dating analyses.

In addition, we constructed an organismal phylogeny restricted to the 62 mycorrhizal fungi. Orthologous genes among the fungi were identified using FastOrtho with the parameters set to 50% identity, 50% coverage, and inflation 3.0 (ref. ^72^). Protein sequences were downloaded from MycoCosm (mycocosm.jgi.doe.gov). Clusters with single-copy genes were identified and aligned with MAFFT 7.221 (ref. ^73^), ambiguous regions (containing gaps and poorly aligned) were eliminated, and single-gene alignments were concatenated with Gblocks 0.91b (ref. ^74^). A phylogenetic tree was constructed with RAxML 7.7.2 (ref. ^75^), the standard algorithm, the PROTGAMMAWAG model of sequence evolution, and 1000 bootstrap replicates.

### Molecular dating

In order to time calibrate the phylogenies, we used the penalized likelihood algorithm as implemented in r8s (ref. ^76^) with the POWELL optimization. To identify the appropriate smoothing parameters, we performed a cross-validation analysis for each dataset. For the Basidiomycota phylogeny, the fossil *Archaeomarasmius legetti* from the mid-Cretaceous (90-94 Ma)^77^ was used to calibrate the node containing the suborder Marasmiineae, and the fossil of a suilloid ectomycorrhiza from the middle Eocene (50 Mya)^78^ was used to calibrate the node containing the suborders Boletineae, Paxillineae, Sclerodermatineae, and Suillineae. For the Ascomycota phylogeny, the fossil *Paleopyrenomycites devonicus* from the early Devonian (~400 Ma)^79^ was used to calibrate the split between Pezizomycotina and Saccharomycotina. The Basidiomycota supermatrix consisted of 940,059 sites from 1042 genes. All nodes were recovered with a bootstrap support of 96% or higher. The Ascomycota supermatrix consisted of 1,474,883 sites from 1432 genes. All nodes were recovered with bootstrap support of 100%.

### Analysis of PCWDE evolution

A comprehensive list of CAZyme annotations can be found in Supplementary Data 11 and at the Mycorrhizal Fungi page at the JGI MycoCosm database by following this link: https://mycocosm.jgi.doe.gov/mycocosm/annotations/browser/cazy/summary;-6jBQE?p=Mycorrhizal_fungi.

We investigated the evolution of gene families encoding CAZymes with a particular interest in PCWDEs, which include enzymes active on cellulose (various GH families, AA3_1, LPMO, and CBM1), lignin (AA1_1, AA1_3, AA2_class II POD, AA5_1, DyP, HTP, and OXO), and pectin (CE8, PL, and GH families; see also Supplementary Data 8). We first performed all-vs-all Blast using mpiBlast 1.6.0 (ref. ^80^) for 51 Ascomycota and 61 Basidiomycota proteomes, and clustered proteins into gene families using the Hifix 1.0.5 clustering method, with default parameters resulting in 243,784 and 408,904 clusters, respectively. Identification of PCWDE families was performed based on InterPro (IPR) domains in the similarity-based clustering (see above). IPR data from the JGI website were used to annotate proteins with IPR domains for all species. We considered clusters as PCWDE families if at least 50% of the proteins had the appropriate IPR domain. Further classification of PCWDE families was supported by BLASTP 2.6.0+ search^80^, and validation by the CAZy annotation pipeline^81^. Multiple sequence alignments were inferred using PRANK 140,603 with default settings, and trim-Al 1.4.rev15 was used for removing ambiguously aligned regions (with the argument -gt 0.1). ML gene trees of the identified PCWDE clusters were inferred using FastTree 2.1.10 under the WAG model of protein evolution with gamma-distributed rate heterogeneity. TreeFix 1.1.10 (ref. ^82^) was used for gene tree/species tree reconciliation (-m PROTGAMMAWAG, –alpha 0.001, –niter 300) with the genome-based ML species trees. Gene clusters containing less than four proteins were excluded from the analysis. For the reconstruction of gene duplication and loss history of PCWDEs, we used the COMPARE pipeline^54^. Orthogroups were identified, and duplications and losses were inferred for each enzyme group across species trees based on Dollo parsimony. The ancestral gene copy numbers for every internal node were calculated by summing the mapped gains and losses over the species tree.

Class I and class II PODs were also curated by the RedoxiBase database (http://peroxibase.toulouse.inra.fr) to distinguish between LiP, versatile PODs, MnP, or atypical Class II PODs. The latter PODs have been annotated as basidiomycete sub-class B (CIIBB) or sub-class C (CIIBC)^83^.

### Phylostratigraphy

Phylogenetic ages of ectomycorrhiza-induced genes were assigned based on the most phylogenetically distant orthologous gene in the containing gene family, using a phylostratigraphic approach^84^. Ectomycorrhiza-induced transcripts were retrieved from RNA-seq-based transcriptome profilings: *Amanita muscaria*, GSE63867; *Cenococcum geophilum*, GSE83909; *Hebeloma cylindrosporum*, GSE63868; *P. involutus*, GSE63924; *Piloderma croceum*, GSE63925; *T. magnatum*, GSE116692; *A. macrosclerotiorum*, SRP130276 and SRP130279-82; *L. bicolor*, SRP164436-38, SRP164526, SRP164559, and SRP164564; *Pisolithus microcarpus*, SRP122806, SRP122812, SRP122818, SRP122826, SRP122829, and SRP122850; and *T. matsutake*, SRP103258). Details of the RNA-seq experiments are described on Gene Expression Omnibus (GEO), NCBI. Note that this pooled set of ectomycorrhiza-induced genes represent a heterogeneous set of symbiosis-induced transcripts from ectomycorrhizal roots from different fungal-tree associations sampled at different stages of development, from early to late stage of symbiosis establishment. We selected those having an expression fold change (FC) > 5 and FC > 2 (Supplementary Data 9). The phylogenetic trees of the Asco- and Basidiomycota were divided into phylostrata, based on the phylogenetic distance in terms of internal nodes from the species being examined. Each phylostratum corresponded to an ancestral species; genes were assigned to phylostrata based on the species distribution of orthogroups the studied genes belong to. Using this method, the ages of upregulated genes were defined and mapped to species trees. We thoroughly selected a subset of the sequenced genomes of species belonging to either the Ascomycota or the Basidiomycota. Running COMPARE on the present set of 135 Glomeromycotina, Ascomycota, and Basidiomycota genomes separately would have been prohibitively time consuming involving disproportionate costs.

### Comparative analyses and annotation of functional categories

Statistics of JGI genome assemblies (i.e., N50, number of genes and scaffolds, and genome size) were obtained from Mycocosm (mycocosm.jgi.doe.gov). Genome completeness with single-copy orthologues was calculated using BUSCO v3.0.2, with odb9 using default parameters^85^. Secretomes were predicted as described in Pellegrin et al.. We compared the different categories of secreted proteins (e.g., CAZymes, lipases, and proteases) according to their lifestyle. Note that the comparisons of genomes are based on analyses of haploid genomes. *P* values were estimated from nonparametric multiple comparisons based on the generalized Campbell and Skillings’ procedure with the function *gao_cs* in R package *nparcomp*^86^. Distributions with the mean of the total count and statistically significant ecological groups (lifestyles; *P* < 0.05) were determined. Genomic features (e.g., genome size and TE coverage) were statistically compared between fungal lifestyles based on the GLS procedure with the Brownian motion model for random evolution, using R package *nlme* with the function *gls*^87^. Output files generated were combined and visualized with a series of custom R scripts, Proteomic Information Navigated Genomic Outlook (PRINGO), incorporating R graphic packages *ggplot2*, *ggtree*, *egg*, and *ggpubr*^88–91^. The coverage of TEs in genomes was calculated and visualized, using a custom pipeline named Transposon Identification Nominative Genome Overview (TINGO)^27^.

We determined the proportion of variances in genomic features explained by fungal ecological groups, and phylogenetic distances of 61 and 51 species from Basidiomycota and Ascomycota, respectively. Phylogenetic trees were converted into evolutionary distances of species with R package *ape*^92^. Genomic features (i.e., TE coverage, genome size, and predicted secreted proteins) were converted into numerical distances for PERMANOVA according to the workflow using R package *vegan*^93,94^. Significantly different variables were examined (*P* value <0.05; PERMANOVA; variables ~ phylogeny + ecology). Significant differences among the fungal ecological groups were tested using pair-wise PERMANOVA with R package RVAideMemoire^95^.

Since the 5′ and 3′ LTR of LTR retrotransposons are identical upon insertion, we estimated their time since insertion using the number of substitutions that occur between the two LTRs as described in Castanera et al.^96^. The list of softwares and R packages is provided in Supplementary Dataset 15. 

### Reporting summary

Further information on research design is available in the Nature Research Reporting Summary linked to this article.

## Supplementary information

Supplementary Information

Peer Review

Reporting Summary

Description of Additional Supplementary Files

Supplementary Dataset 1

Supplementary Dataset 2

Supplementary Dataset 3

Supplementary Dataset 4

Supplementary Dataset 5

Supplementary Dataset 6

Supplementary Dataset 7

Supplementary Dataset 8

Supplementary Dataset 9

Supplementary Dataset 10

Supplementary Dataset 11

Supplementary Dataset 12

Supplementary Dataset 13

Supplementary Dataset 14

Supplementary Dataset 15

Supplementary Dataset 16

## Data Availability

Genome assemblies and annotations for the organisms used in this study are available via the JGI fungal genome portal MycoCosm (mycocosm.jgi.doe.gov). In addition, the newly sequenced genome assemblies and annotations have been deposited to GenBank (see Supplementary Data 1 for accession codes/BioProjects). The complete transcriptome datasets are available at the GEO at the National Center for Biotechnology Information (http://www.ncbi.nlm.nih.gov/geo/). The accession codes for accessing the data deposited at GEO database are provided in the “Methods/Phylostratigraphy” section and in the caption of Supplementary Fig. 6. All other data supporting the findings of this study are available within the article and its Supplementary information files or are available from the corresponding authors upon request. Source data are provided with this paper.

## Source data

Source Data

## Code availability

PRINGO R scripts are deposited at GitHub: https://github.com/ShingoMiyauchi/PRINGO. The program COMPARE is also available at Github: https://github.com/laszlognagy/COMPARE.
