# Supplementary material for: Large-scale genome sequencing of mycorrhizal fungi provides insights into the early evolution of symbiotic traits
Source: Nat Commun. 2020 Oct 12;11:5125. doi: 10.1038/s41467-020-18795-w (PMC7550596; doi:10.1038/s41467-020-18795-w)
Supplement: Supplementary file 1 — Supplementary Information [file 41467_2020_18795_MOESM1_ESM.pdf]

Supplementary information

Large-scale genome sequencing of mycorrhizal fungi provides insights into the early evolution of symbiotic traits

Miyauchi *et al.*

Supplementary Methods

Genome sequencing and assembly

The 29 new genomes for the fungi reported in this study were sequenced using several different sequencing platforms described below, assembled with several assembly tools listed in Supplemental Data 11, and annotated with the JGI Annotation Pipeline [1,2] aided by RNA sequences when available.

Ten genomes (*Acephala*, *Ceratobasidium*, *Clavulina*, *Gyrodon*, *Marasmius*, *Paxillus*, *Thelephora ganbajun*, *Tricholoma*, *Wilcoxina*, and *Xerocomus*) were sequenced using Illumina alone using 300 bp Illumina Regular Fragment libraries. For these, 100 ng of DNA was sheared to 300 bp using the Covaris LE220 and size selected using SPRI beads (Beckman Coulter). The fragments were treated with end-repair, A-tailing, and ligation of Illumina compatible adapters (IDT, Inc) using the KAPA-Illumina library creation kit (KAPA Biosystems). The prepared libraries were quantified using KAPA Biosystem's next-generation sequencing library qPCR kit and run on a Roche LightCycler 480 real-time PCR instrument. The quantified libraries were then multiplexed with other libraries, and the pool of libraries was then prepared for sequencing on the Illumina HiSeq sequencing platform utilizing a TruSeq paired-end cluster kit, v4, and Illumina's cBot instrument to generate a clustered flow cell for sequencing. Sequencing of the flow cell was performed on the Illumina HiSeq2500 sequencer using HiSeq TruSeq SBS sequencing kits, v4, following a 2x150 indexed run recipe.

For *Ceratobasidium*, *Gyrodon*, *Marasmius*, *Tricholoma*, and *Wilcoxina*, additional 4kb CLRS Illumina Regular Long Mate-Pair (LMP) libraries were created; 5-6 µg of DNA was sheared using the Covaris g-TUBE™ (Covaris) and gel size selected for 4kb. The sheared DNA was treated with end repair and ligated with biotinylated adapters containing loxP. The adapter ligated DNA fragments were circularized via recombination by a Cre excision reaction (NEB). The circularized DNA templates were then randomly sheared using the Covaris LE220 (Covaris).The sheared fragments were treated with end repair and A-tailing using the KAPA-Illumina library creation kit (KAPA Biosystems) followed by immobilization of mate pair fragments on streptavidin beads (Invitrogen). Illumina compatible adapters (IDT, Inc) were ligated to the mate pair fragments and 8 cycles of PCR was used to enrich for the final library (KAPA Biosystems). The libraries were then treated as with the above Regular Fragment libraries, except for following a 2x100 indexed run recipe. The *Tricholoma* procedure used additional LMP libraries that varied from the others in insert size (4.5kb and 8kb) and/or in amount of source DNA (15 µg); these were otherwise treated the same as the others.

Fifteen genomes (*Amanita*, *Boletus* [both strains], *Cantharellus*, *Gautieria*, *Hysterangium*, *Kalaharituber*, *Lactarius*, *Mycena*, *Russula* [both species], *Terfezia*, *Thelephora terrestris*, *Tirmania*, and *Trichophaea*) were sequenced using Pacific Biosciences sequencing alone. With the exceptions of *Cantharellus* and *Mycena*, all genomes were sequenced using PacBio >10kb with AMPure Bead Size Selection and Tubes. Unamplified libraries were generated using Pacific Biosciences standard template preparation protocol for creating >10kb libraries. Five µg of gDNA was used to generate each library and the DNA was sheared using Covaris g-Tubes to generate sheared fragments of >10kb in length. The sheared DNA fragments were then prepared using Pacific Biosciences SMRTbell template preparation kit, where the fragments were treated with DNA damage repair, had their ends blunt-ended, and 5' phosphorylated. Pacific Biosciences hairpin adapters were then ligated to the fragments to create the SMRTbell template for sequencing. The SMRTbell templates were then purified using exonuclease treatments and size-selected using AMPure PB beads. For *Cantharellus*, *Mycena*, and an additional *Boletus edulis* BED1 library, the library method differed in that the SMRTbell templates were size-selected using the Sage Science BluePippin instrument with a 4kb lower cutoff.

For *Amanita*, *Boletus edulis* Přilba, both *Russula* sp., *T. terrestris*, and *Tirmania*, PacBio Sequencing primer was then annealed to the SMRTbell template library and sequencing polymerase was bound to them using Sequel Binding kit 2.0. The prepared SMRTbell template libraries were then sequenced on a Pacific Biosystem's Sequel sequencer using v3 sequencing primer, 1M v2 SMRT cells, and Version 2.1 v2 sequencing chemistry with 6 & 10 hour sequencing movie run times. For *Cantharellus*, *Kalaharituber*, *Lactarius*, *Mycena*, *Terfezia*, and *Trichophaea*, sequencing primer was then annealed to the SMRTbell templates and Version P6 sequencing polymerase was bound to them. The prepared SMRTbell template libraries were then sequenced on a Pacific Biosciences RSII sequencer using Version C4 chemistry and 4 hour sequencing movie run times. The procedure for *B. edulis* BED1 and *Hysterangium* differed from that of *Cantharellus* above only in using Version C2 chemistry and 3 hour sequencing movie run times.

Four genomes (*Cortinarius*, *Hydnum*, *Melanogaster*, and *Ramaria*) were sequenced by hybrid approaches using both Illumina and PacBio. In all cases except for *Cortinarius*, 300bp Illumina Regular Fragment libraries were generated and sequenced as above. The *Cortinarius* procedure differed in its creation of a 270bp Illumina Regular Fragment library, which was sequenced using a TruSeq SBS sequencing kit, v3. The *Ramaria* procedure involved the additional creation of 4kb and 8kb CLRS Illumina Regular LMP libraries with methods identical to that above, except for the use of 10 µg of DNA for the 8kb library. All 4 genomes were additionally sequenced using the same PacBio library and sequencing method as described for *Hysterangium* above.

The five genomes with both Illumina Fragment and LMP libraries (*Ceratobasidium*, *Gyrodon*, *Marasmius*, *Tricholoma*, and *Wilcoxina*) were assembled with ALLPATHS-LG (APLG) [3]. For the five genomes with Illumina Fragment libraries only (*Acephala*, *Clavulina*, *Paxillus*, *T. ganbajun*, and *Xerocomus*), LMP reads were simulated by an initial assembly of the Illumina Fragment library using Velvet [4], which were then used to improve the final APLG assembly. The 15 genomes sequenced with PacBio only were assembled using FALCON [5], with the exception of *Hysterangium*, for which the Celera Assembler [6]

was used. For the remaining four genomes sequenced using a hybrid Illumina-PacBio approach (*Cortinarius*, *Hydnum*, *Melanogaster*, and *Ramaria*), the Illumina Fragment and LMP reads were assembled using APLG, and the resulting assemblies were patched with PacBio reads using PBJelly [7].

Transcriptome sequencing and assembly for genome annotations

Sequenced transcriptomes of free-living mycelium were used to assess the completeness of genome assemblies and to seed and assess genome annotations. All transcriptomes were sequenced using Illumina RNA-Seq with polyA selection. Except for five species' procedures differing in minor ways and noted below, stranded cDNA libraries were generated using the Illumina Truseq Stranded RNA LT kit. mRNA was purified from 1 µg of total RNA using magnetic beads containing poly-T oligos. mRNA was fragmented and reversed transcribed using random hexamers and SSII (Invitrogen) followed by second strand synthesis. The fragmented cDNA was treated with end-pair, A-tailing, adapter ligation, and 8 or 10 cycles of PCR. The prepared libraries were quantified using KAPA Biosystem's next-generation sequencing library qPCR kit and run on a Roche LightCycler 480 real-time PCR instrument. The quantified libraries were then multiplexed with other libraries, and the pool of libraries was then prepared for sequencing on the Illumina HiSeq sequencing platform utilizing a TruSeq paired-end cluster kit, v4, and Illumina's cBot instrument to generate a clustered flow cell for sequencing. Sequencing of the flow cell was performed on the Illumina HiSeq2500 sequencer using HiSeq or TruSeq SBS sequencing kits, v4, following a 2x150 indexed run recipe.

The *Tirmania*, *T. terrestris*, and *Russula emetica* transcriptome methods differed by sequencing the libraries on the Illumina NovaSeq sequencer using NovaSeq XP v1 reagent kits, S4 flow cell. The procedure for the *Cortinarius* and *Tricholoma* transcriptomes differed from the others in that the pool of libraries was prepared utilizing a TruSeq paired-end cluster kit, v3, and in that sequencing of the flow cell was performed on the Illumina HiSeq2000 sequencer using a TruSeq SBS sequencing kit, v3, following a 2x100 indexed run recipe. No RNA samples were available for *Hysterangium* or *Melanogaster* assembly or annotation.

For nine transcriptomes (*Acephala*, *Amanita*, *B. edulis* Přilba, both *Russula* sp., *Terfezia*, *T. terrestris*, *Tirmania*, and *Trichophaea*), the reads were assembled into RNA contigs using Trinity [8]. For the remaining 18 transcriptomes, the reads were assembled into RNA contigs using Rnnotator [9].

Genome Annotation

Each genome was annotated using the JGI Annotation Pipeline, which detects and masks repeats and transposable elements, predicts genes, characterizes each conceptually translated protein with sub-elements such as domains and signal peptides, chooses a best gene model at each locus to provide a filtered working set, clusters the filtered sets into draft gene families, ascribes functional descriptions (such as GO terms and EC numbers, and creates a JGI genome portal with tools for public access and community-driven curation of the annotation [1,2]. Note that the comparisons of genomes are based on analyses of haploid genomes.

Transcriptome datasets for phylostratigraphy and gene expression analysis

Gene expression data used for the analyses of phylostratigraphy and phylogenetic conservation of ectomycorrhiza-induced genes were extracted from previously published RNA-Seq experiments. Details of the RNA-seq experiments are described at the NCBI Gene Expression Omnibus (GEO). Accession code numbers are as follows: *Amanita muscaria*, GSE63867; *Cenococcum geophilum*, GSE83909; *Hebeloma cylindrosporum*, GSE63868; *P. involutus*, GSE63924; *Piloderma croceum*, GSE63925; *Tuber magnatum,* GSE116692; *A. macrosclerotiorum*, SRP130276, SRP130279-82; *L. bicolor*, SRP164436-38, SRP164526, SRP164559, SRP164564; *Pisolithus microcarpus,* SRP122806, SRP122812, SRP122818, SRP122826, SRP122829, SRP122850 and *T. matsutake*, SRP103258). For CAZymes gene expression, data were also extracted from previously published RNA-Seq experiments. Details can be found at the NCBI Gene Expression Omnibus in [12] for *L. bicolor*, *A. muscaria*, *H. cylindrosporum*, *P. croceum* and *P. involutus* (superseries GSE63947), in [13] for *T. magnatum* (GSE116692), in [14] for *C. geophilum* (GSE83909) and in [15] for *M. bicolor* (GSE107845*)*, *R. ericae* (GSE107647) and *O. maius* (GSE63922*)*. RNA-Seq data for *A. macrosclerotiorum* were submitted to NCBI-SRA with accession numbers SRP130276 and SRP130279-82.

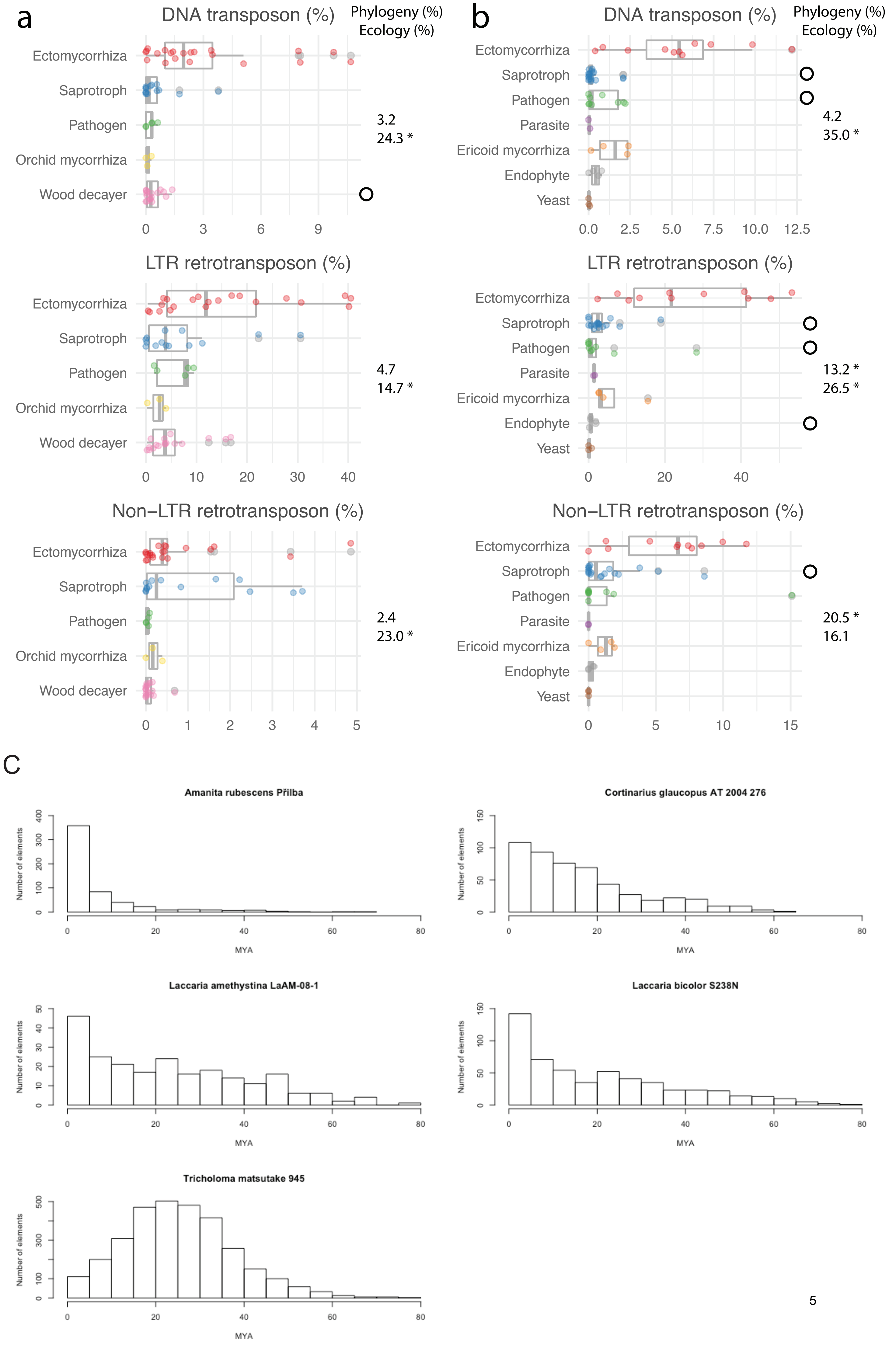

a
DNA transposon (%)
Phylogeny (%)
Ecology (%)
Ectomycorrhiza
Saprotroph
Pathogen
Orchid mycorrhiza
Wood decayer
3.2
24.3 *
LTR retrotransposon (%)
4.7
14.7 *
Non–LTR retrotransposon (%)
2.4
23.0 *
b
DNA transposon (%)
Phylogeny (%)
Ecology (%)
Ectomycorrhiza
Saprotroph
Pathogen
Parasite
Ericoid mycorrhiza
Endophyte
Yeast
4.2
35.0 *
LTR retrotransposon (%)
13.2 *
26.5 *
Non–LTR retrotransposon (%)
20.5 *
16.1
C
Amanita rubescens Přilba
Cortinarius glaucopus AT 2004 276
Laccaria amethystina LaAM-08-1
Laccaria bicolor S238N
Tricholoma matsutake 945
Number of elements
MYA

Supplementary Fig. 1a. **Genome coverage of the main transposon types among ecological guilds and age of *Gypsy* LTR-retrotransposon insertions** (**a**). Basidiomycota. The number of species per ecological guilds is as follows: ectomycorrhiza (n = 32), orchid mycorrhiza (n = 3), pathogens (n = 5), soil/litter saprotrophs (n = 14), wood decayers (n = 17). (**b**). Ascomycota. Ectomycorrhiza (n = 13), ericoid mycorrhiza (n = 4), endophytes (n = 4), parasites (n = 2), pathogens (n = 9), saprotrophs (n = 18) and yeasts (n = 3). TE coverage: The percentage of transposable elements covering the genomes. The boxplots represent median, upper, lower quartiles with the whiskers showing minimal and maximal values and outliers in circles. The small dots show single observations. Asterisks indicate (i) significantly different phylogenetic distances of species and ecological guilds (lifestyles) (PERMANOVA: p-value < 0.05; Genomic features ~ Phylogeny + Ecology; see Supplementary Data 3 for degrees of freedom, F-test, and R^2). Open circles indicate significantly different ecological guilds compared to ectomycorrhizal fungi (pair-wise PERMANOVA: FDR adjusted p-value < 0.05; see Supplementary Data 3 for details of degrees of freedom, F-test, and R^2). Please note that the endophyte guild in Basidiomycota was excluded as it only contains a single species. (**c) Age distribution of *Gypsy* LTR-retrotransposon insertions in five selected ectomycorrhizal fungi in Agaricales.** The insertion ages of all intact LTR-retrotransposons (carrying both Long Terminal Repeats) were estimated based on the nucleotide divergence of LTR-LTR divergence using the approach described in [10] and the fungal substitution rate of 1.05×10^{-9} nucleotides per site per year. Counts of number of insertions in 5 million years (MYA) bins are shown. Source data are provided as a Source Data Supplementary figures 1a and 1b.

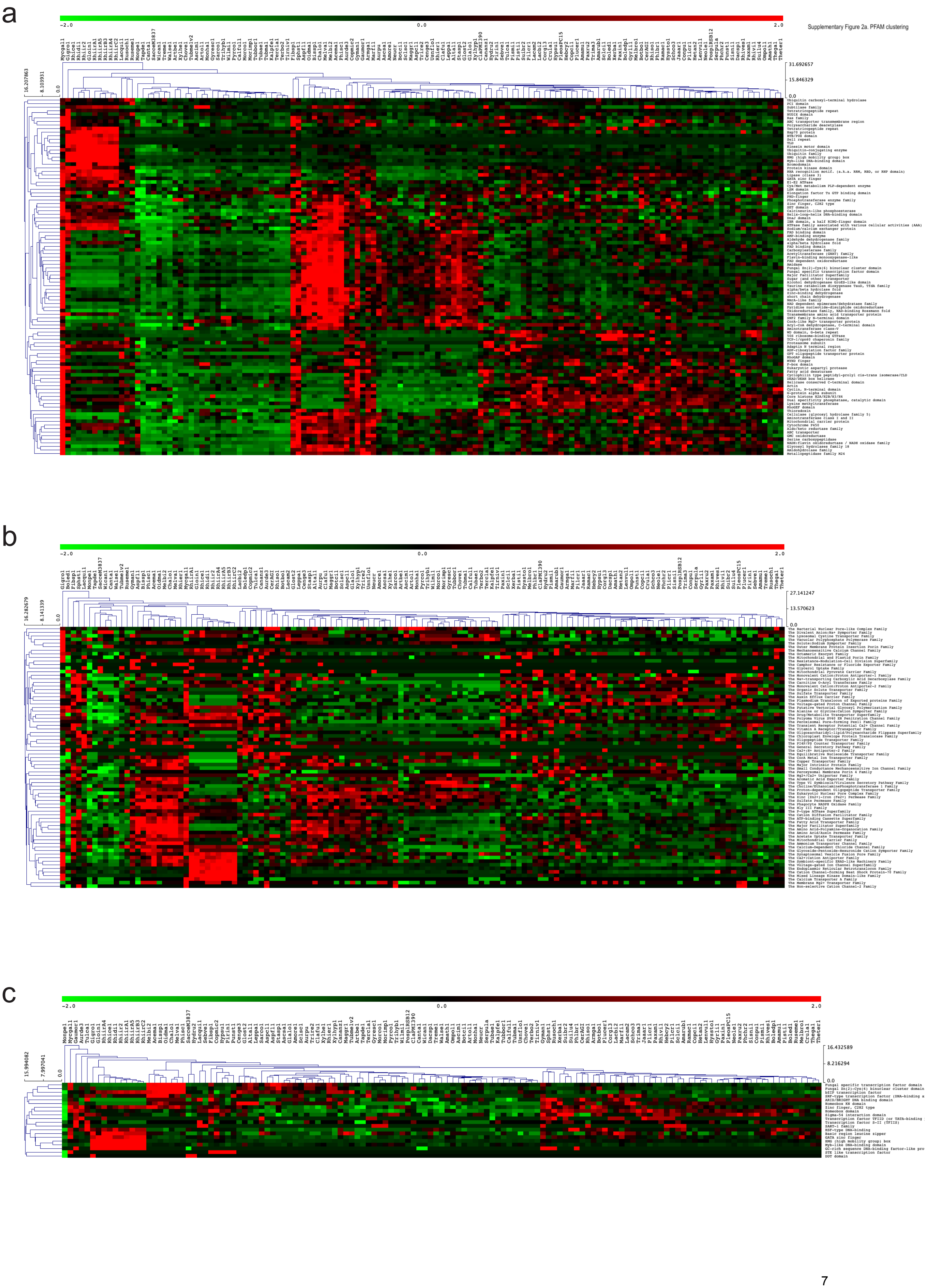

a
Supplementary Figure 2a. PFAM clustering
-2.0
0.0
2.0
31.692657
15.846329
0.0
16.207863
8.103931
b
27.141247
13.570623
16.282679
8.141339
c
16.432589
8.216294
15.994082
7.997041

Supplementary Fig. 2a. Distribution of Pfam protein domains encoded by the 135 genomes. Presence and abundance of the different Pfam domain-containing proteins in the 135 genomes of Glomeromycota, Ascomycota and Basidiomycota species analyzed in this study. The heat map depicts absolute Pfam domain counts in each of the sampled genomes, according to the colour scale (only the top most frequent 100 domains are shown). The abundance values were then transformed into z-scores, which are measure of relative enrichment (red) and depletion (green); the hierarchical clustering was done with a Euclidian distance metric and average linkage clustering method. The data were visualised and clustered using MultiExperiment VIEWER (http://www.tm4.org/mev.html). Source data are provided as a Source Data Supplementary figure 2.

Supplementary Fig. 2b. Presence and abundance of genes coding for membrane transporters in the 135 genomes of Glomeromycota, Ascomycota and Basidiomycota species analyzed in this study. The heat map depicts absolute numbers of membrane transporter genes counts in each of the sampled genomes, according to the colour scale (only the top most frequent 75 transporter categories are shown). The abundance values were then transformed into z-scores, which are measure of relative enrichment (red) and depletion (green); the hierarchical clustering was done with a Euclidian distance metric and average linkage clustering method. The data were visualised and clustered using MultiExperiment VIEWER (http://www.tm4.org/mev.html). Source data are provided as a Source Data Supplementary figure 2.

Supplementary Fig. 2c. Presence and abundance of genes coding for transcriptional factors in the 135 genomes of Glomeromycota, Ascomycota and Basidiomycota species analyzed in this study. The heat map depicts absolute numbers of membrane transporter genes counts in each of the sampled genomes, according to the colour scale (only the top most frequent 20 transcription factor categories are shown). The abundance values were then transformed into z-scores, which are measure of relative enrichment (red) and depletion (green); the hierarchical clustering was done with a Euclidian distance metric and average linkage clustering method. The data were visualised and clustered using MultiExperiment VIEWER (http://www.tm4.org/mev.html). Source data are provided as a Source Data Supplementary figure 2.

a

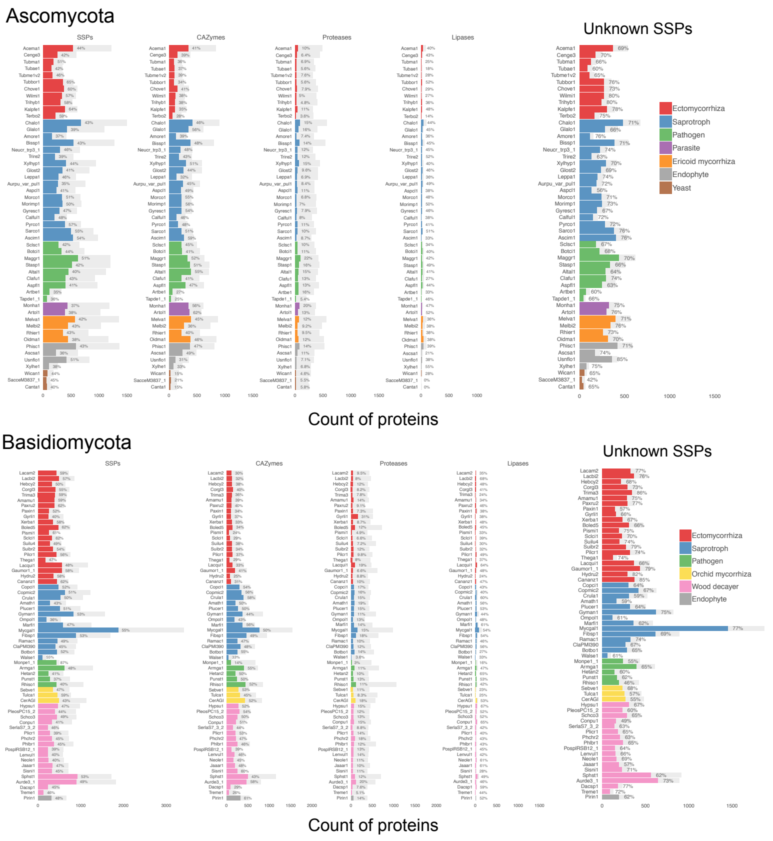

Ascomycota
SSPs
CAZymes
Proteases
Lipases
Unknown SSPs
Ectomycorrhiza
Saprotroph
Pathogen
Parasite
Ericoid mycorrhiza
Endophyte
Yeast
Count of proteins
Basidiomycota
SSPs
CAZymes
Proteases
Lipases
Unknown SSPs
Ectomycorrhiza
Saprotroph
Pathogen
Orchid mycorrhiza
Wood decayer
Endophyte
Count of proteins

b

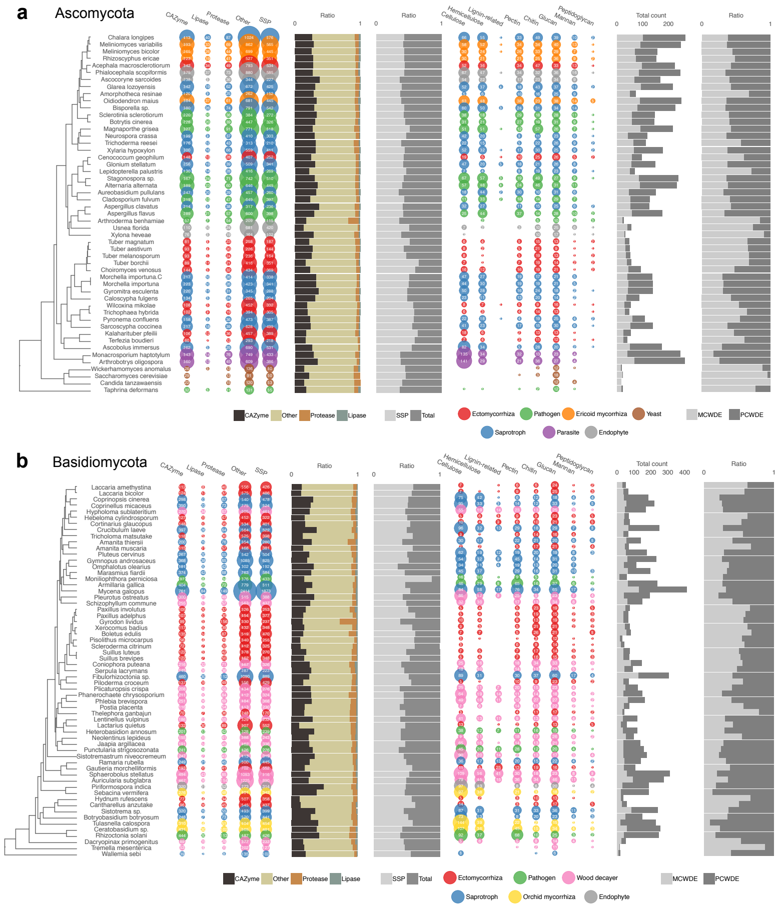

a
Ascomycota
Ratio
Total count
CAZyme
Other
Protease
Lipase
SSP
Total
Ectomycorrhiza
Pathogen
Ericoid mycorrhiza
Yeast
Saprotroph
Parasite
Endophyte
MCWDE
PCWDE
b
Basidiomycota
Ratio
Total count
CAZyme
Other
Protease
Lipase
SSP
Total
Ectomycorrhiza
Pathogen
Wood decayer
Saprotroph
Orchid mycorrhiza
Endophyte
MCWDE
PCWDE

c

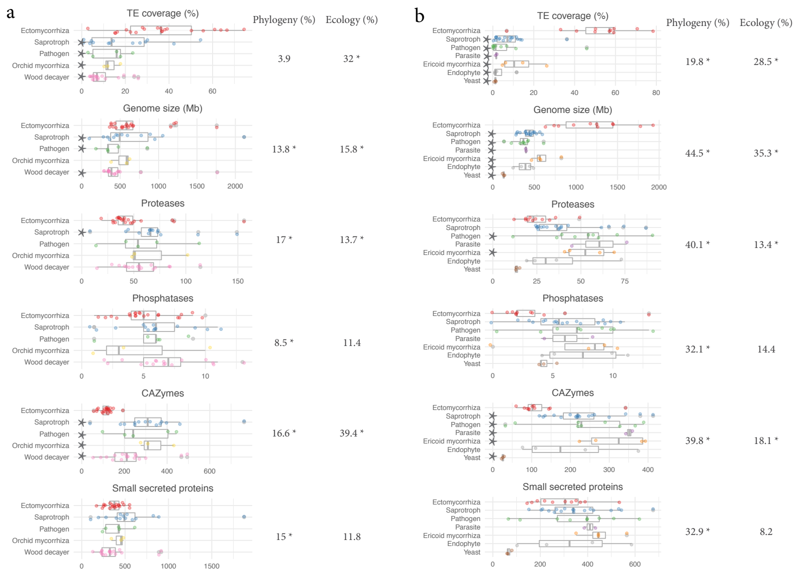

a
TE coverage (%)
Phylogeny (%)
Ecology (%)
Genome size (Mb)
Proteases
Phosphatases
CAZymes
Small secreted proteins
b
TE coverage (%)
Phylogeny (%)
Ecology (%)
Genome size (Mb)
Proteases
Phosphatases
CAZymes
Small secreted proteins

d

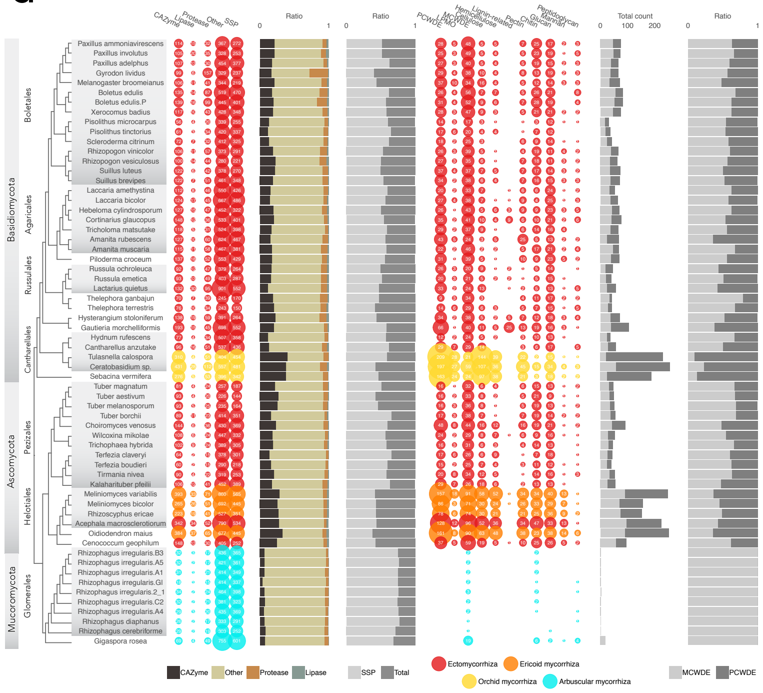

Basidiomycota
Boletales
Agaricales
Russulales
Cantharellales
Ascomycota
Pezizales
Helotiales
Mucoromycota
Glomerales
Ratio
Total count
CAZyme
Other
Protease
Lipase
SSP
Total
Ectomycorrhiza
Ericoid mycorrhiza
Orchid mycorrhiza
Arbuscular mycorrhiza
MCWDE
PCWDE

Supplementary Fig. 3a. **Proportion of predicted secreted proteins in the protein repertoire for 51 Ascomycota and 61 Basidiomycota.** The selected fungi are grouped based on their lifestyles. Colour bars indicate the count of genes coding for secreted proteins. The percentage of secreted proteins is shown. Gray bars represent the count of all proteins (secreted + non-secreted) in the categories. Total count of three categories of secreted proteins (CAZymes, lipases, proteases) and the subcategory corresponding to SSPs. Note that the proportion of SSPs is compared to the total count of secreted proteins. See Supplementary Data 6 and 7 for detailed counts. Unknown SSPs: Percentage of SSPs with no know function among total SSPs. Identified functions for SSPs are listed in Supplementary Data 12. The total number of CAZymes is based on the annotations from the CAZy database (http://www.cazy.org) (Supplementary Data 11). Source data are provided as a Source Data Supplementary figure 3a.

Supplementary Fig. 3b. **Predicted secretomes of 51 Ascomycota and 61 Basidiomycota**. The species are ordered according to time-calibrated maximum-likelihood phylogenies. The first bubble plot (next to the tree) shows the number of secreted genes for CAZymes, lipases, proteases, and others (i.e. all secreted proteins not in these first three groups). The SSP group is a subcategory showing the number of small secreted proteins. The size of the bubbles corresponds to the number of genes. The fungal species are coloured according to their lifestyle. The first bar plots in the middle represent the proportion of CAZymes, lipases, proteases, to all secreted proteins (left); and the proportion of SSPs among the combined secretomes (right). The second bubble plot (between two bar plots) shows the number of enzymes acting on cellulose, hemicellulose, lignin- and polyphenolic-related compounds and pectin (plant cell walls); chitin, glucans, mannans (fungal cell walls) and peptidoglycans (bacterial cell walls). The second bar plots (far right) show the total count of genes including plant cell wall degrading enzymes (PCWDEs) and microbial cell wall degrading enzymes (MCWDEs) (left); and the proportion of PCWDEs to MCWDEs (right). See also Supplementary Data 6 a-d for the corresponding gene counts. See Supplementary Data 7 a-c for the PCWDEs and MCWDE comparison. See Supplementary Fig. 3d for the secretome profiles of 62 mycorrhizal fungi. Source data are provided as a Source Data Supplementary figure 3b.

Supplementary Fig. 3c. **Distribution of genomic features among the ecological guilds.** (**a**). Basidiomycota. The number of species per ecological guilds is as follows: ectomycorrhizal fungi (n = 32), orchid mycorrhizal fungi (n = 3), pathogens (n = 5), soil/litter saprotrophs (n = 14), and wood decayers (n = 17). (**b**). Ascomycota. The number of species per ecological guilds is as follows: ectomycorrhizal fungi (n = 13), ericoid mycorrhizal fungi (n = 4), endophytes (n = 4), parasites (n = 2), pathogens (n = 9), soil/litter saprotrophs (n = 18) and yeast (n = 3). TE coverage: The percentage of transposable elements in the genome assemblies. Genome size, genomes size in megabases. Proteases: number of secreted proteases. Phosphatases: number of secreted phosphatases. CAZymes: number of secreted CAZymes. Small secreted proteins: number of small secreted proteins (size < 300 amino acids). The boxplots represent median, upper, lower quartiles with the whiskers showing minimal and maximal values and outliers in circle. Asterisks indicate (i) significantly different phylogenetic distances of species and ecological guilds. (PERMANOVA: p-value < 0.05; Genomic features ~ Phylogeny + Ecology; see Supplementary Data 3 for degrees of freedom, F-test, and R^2), (ii) significantly different ecological guilds compared to ectomycorrhizal fungi (pair-wise PERMANOVA: FDR adjusted p-value < 0.05; see Supplementary Data 3 for details of degrees of freedom, F-test, and R^2). Please note that the endophytic guild in Basidiomycota was excluded as it only contains a single species. Source data are provided as a Source Data Supplementary figure 3c.

Supplementary Fig 3d. **Predicted secretome profiles for 62 mycorrhizal fungi**. A maximum-likelihood phylogeny with fungal orders is shown on the left panel. The first bubble plot (next to the tree) shows the number of genes for secreted CAZymes, lipases, proteases, SSP and others (i.e. all secreted proteins not in these first three groups). The bubble size is proportional to the number of genes. Taxa are colour-coded according to the fungal lifestyle (see bottom panel). The first bar plots represent the proportion of secreted CAZymes, lipases, proteases and other secreted proteins in the total secretome (left) and the proportion of SSPs in the total secretome (right). The second bubble plot (between two bar plots) shows the number of genes for secreted CAZymes acting on plant cell wall polysaccharides (cellulose, hemicellulose, lignin-related compounds and pectin), fungal cell walls (chitin, glucans, mannans) and bacterial cell walls (peptidoglycans). The second bar plots (far right) shows the total count of genes including PCWDEs and MCWDEs (left); and the PCWDEs/MCWDEs ratio (right). See Supplementary Data 7 d-f. MCWDEs includes enzymes acting on fungal and bacterial cell walls. Source data are provided as a Source Data Supplementary figure 3d.

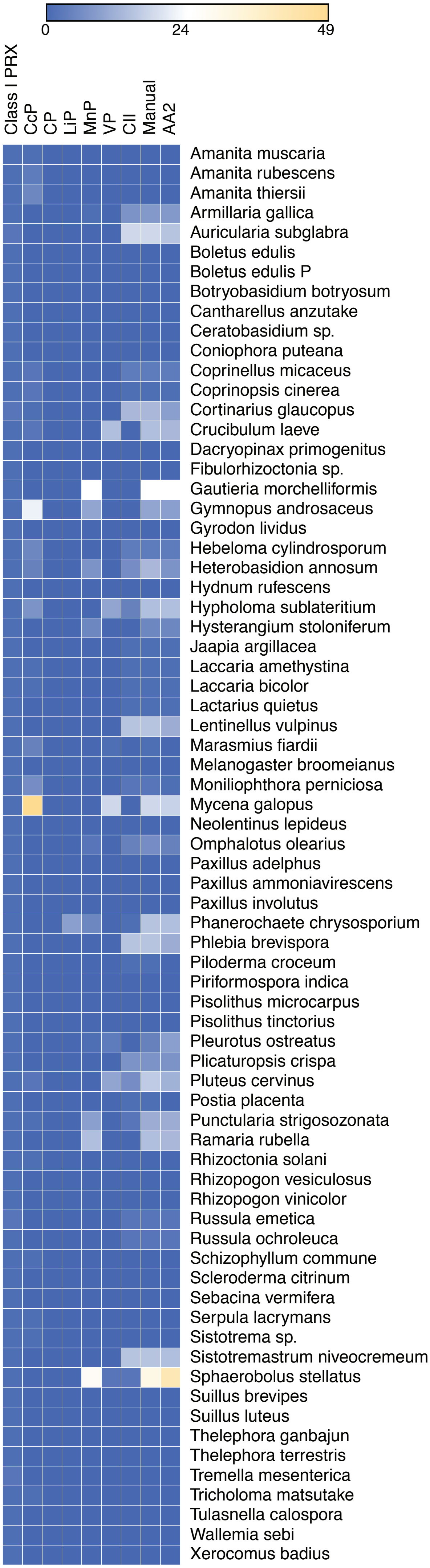
0
24
49
Class I PRX
CcP
CP
LiP
MnP
VP
CII
Manual
AA2
Amanita muscaria
Amanita rubescens
Amanita thiersii
Armillaria gallica
Auricularia subglabra
Boletus edulis
Boletus edulis P
Botryobasidium botryosum
Cantharellus anzutake
Ceratobasidium sp.
Coniophora puteana
Coprinellus micaceus
Coprinopsis cinerea
Cortinarius glaucopus
Crucibulum laeve
Dacryopinax primogenitus
Fibulorhizoctonia sp.
Gautieria morchelliformis
Gymnopus androsaceus
Gyrodon lividus
Hebeloma cylindrosporum
Heterobasidion annosum
Hydnum rufescens
Hypholoma sublateritium
Hysterangium stoloniferum
Jaapia argillacea
Laccaria amethystina
Laccaria bicolor
Lactarius quietus
Lentinellus vulpinus
Marasmius fiardii
Melanogaster broomeianus
Moniliophthora perniciosa
Mycena galopus
Neolentinus lepideus
Omphalotus olearius
Paxillus adelphus
Paxillus ammoniavirescens
Paxillus involutus
Phanerochaete chrysosporium
Phlebia brevispora
Piloderma croceum
Piriformospora indica
Pisolithus microcarpus
Pisolithus tinctorius
Pleurotus ostreatus
Plicaturopsis crispa
Pluteus cervinus
Postia placenta
Punctularia strigosozonata
Ramaria rubella
Rhizoctonia solani
Rhizopogon vesiculosus
Rhizopogon vinicolor
Russula emetica
Russula ochroleuca
Schizophyllum commune
Scleroderma citrinum
Sebacina vermifera
Serpula lacrymans
Sistotrema sp.
Sistotremastrum niveocremeum
Sphaerobolus stellatus
Suillus brevipes
Suillus luteus
Thelephora ganbajun
Thelephora terrestris
Tremella mesenterica
Tricholoma matsutake
Tulasnella calospora
Wallemia sebi
Xerocomus badius

Supplementary Fig. 4. The number of the class II peroxidase coding genes among 135 fungi. Class I PRX: Non-animal peroxidases. CcP: Cytochrome C peroxidase. CP: Catalase peroxidase. Lip: Lignin peroxidase. MnP: Manganese peroxidase. VP: Versatile peroxidase. Other CII: Atypical class II peroxidases. Manual: Number of genes found by manual curation of genomes. AA2: Class II POD found in CAZyme annotations (http://www.cazy.org). See Supplementary Data 6p. Detailed annotations of these peroxidases can be found in the RedoxiBase database (http://peroxibase.toulouse.inra.fr). Source data are provided as a Source Data Supplementary figure 4.

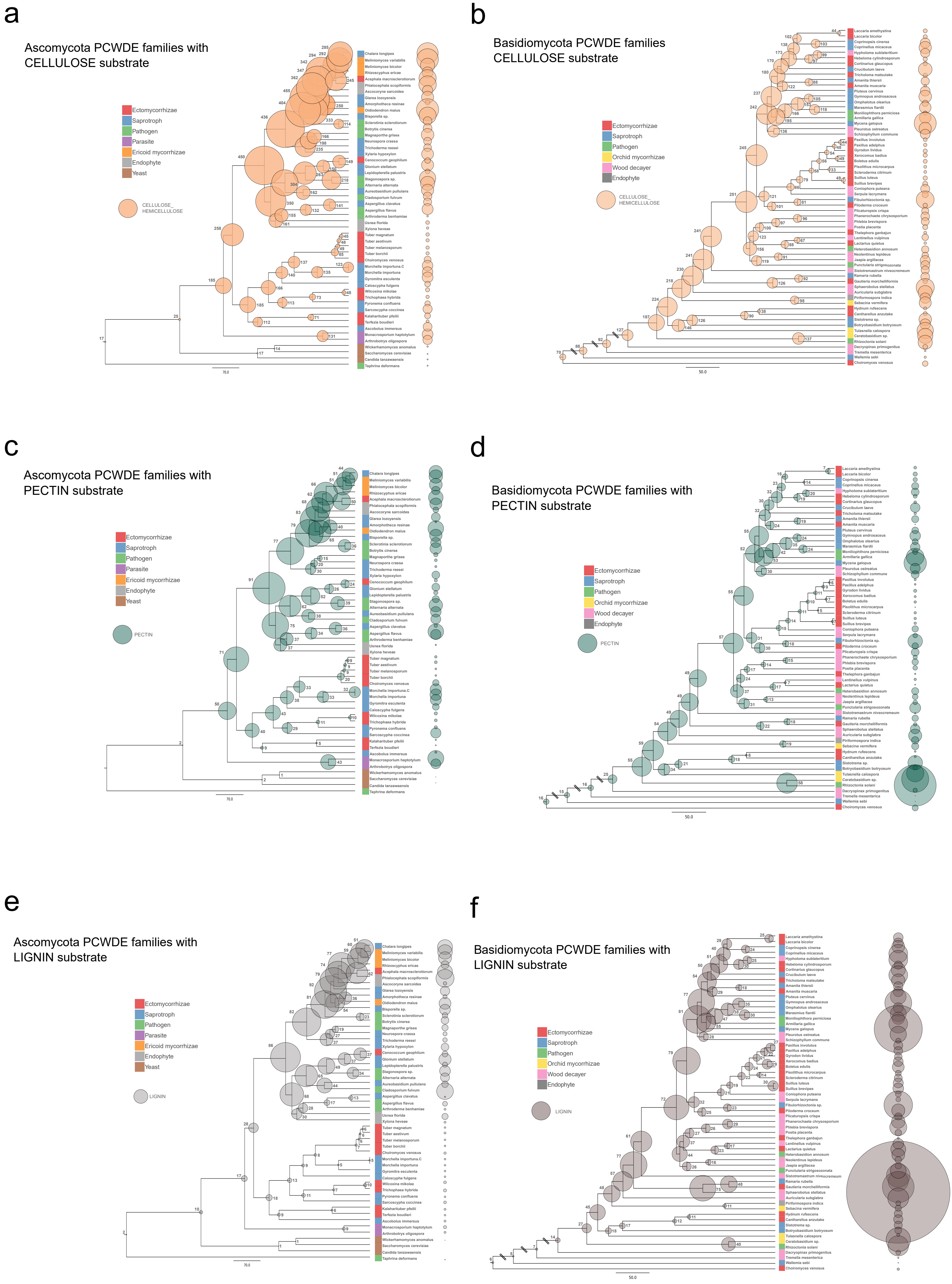
a
Ascomycota PCWDE families with
CELLULOSE substrate
Ectomycorrhizae
Saprotroph
Pathogen
Parasite
Ericoid mycorrhizae
Endophyte
Yeast
CELLULOSE_
HEMICELLULOSE
70.0
b
Basidiomycota PCWDE families
CELLULOSE substrate
Ectomycorrhizae
Saprotroph
Pathogen
Orchid mycorrhizae
Wood decayer
Endophyte
CELLULOSE_
HEMICELLULOSE
50.0
c
Ascomycota PCWDE families with
PECTIN substrate
Ectomycorrhizae
Saprotroph
Pathogen
Parasite
Ericoid mycorrhizae
Endophyte
Yeast
PECTIN
70.0
d
Basidiomycota PCWDE families with
PECTIN substrate
Ectomycorrhizae
Saprotroph
Pathogen
Orchid mycorrhizae
Wood decayer
Endophyte
PECTIN
50.0
e
Ascomycota PCWDE families with
LIGNIN substrate
Ectomycorrhizae
Saprotroph
Pathogen
Parasite
Ericoid mycorrhizae
Endophyte
Yeast
LIGNIN
70.0
f
Basidiomycota PCWDE families with
LIGNIN substrate
Ectomycorrhizae
Saprotroph
Pathogen
Orchid mycorrhizae
Wood decayer
Endophyte
LIGNIN
50.0

Supplementary Fig. 5. Evolutionary gains and losses of genes coding for PCWDEs acting on cellulose (a, b), pectin (c, d) and lignin-related compounds (e, f) for 61 Basidiomycota and 51 Ascomycota. Bubbles with numbers on the phylogenies represent the number of genes for substrate-specific plant cell wall degrading enzyme (PCWDE) families for the ancestral states. The bubble size is proportional to the number of genes. See Supplementary Data 8 for detailed clusters. Source data are provided as a Source Data figure 2.

a

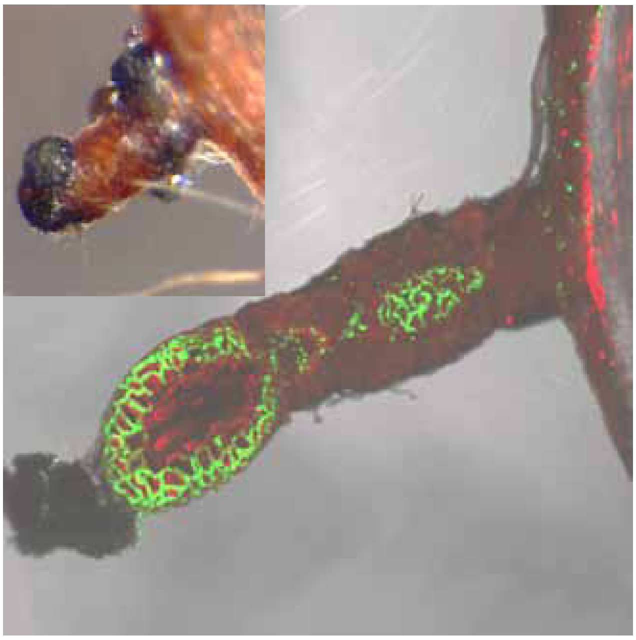

b

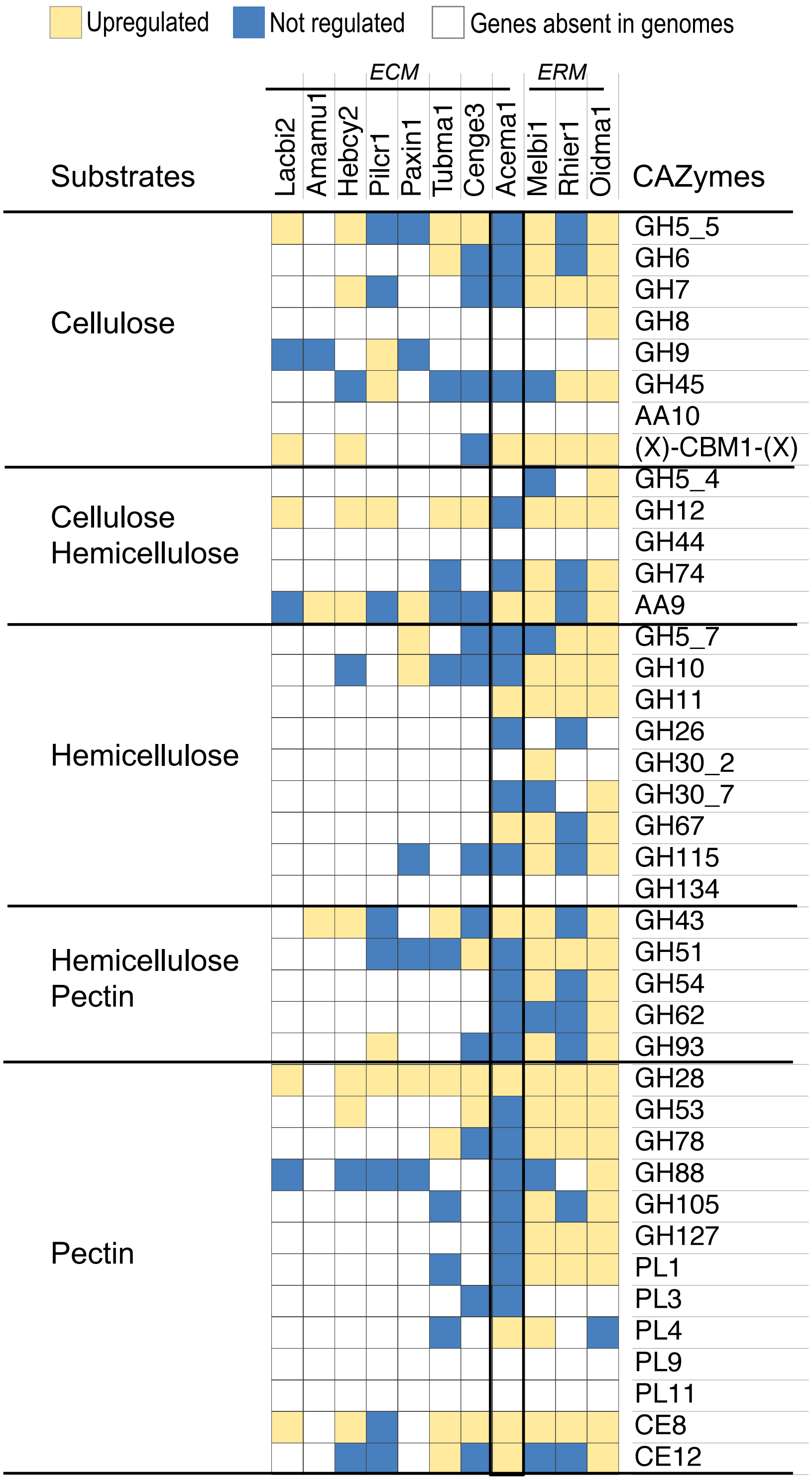

Upregulated
Not regulated
Genes absent in genomes
ECM
ERM
Substrates
Lacbi2
Amamu1
Hebcy2
Pilcr1
Paxin1
Tubma1
Cenge3
Acema1
Melbi1
Rhier1
Oidma1
CAZymes
Cellulose
Cellulose
Hemicellulose
Hemicellulose
Hemicellulose
Pectin
Pectin
GH5_5
GH6
GH7
GH8
GH9
GH45
AA10
(X)-CBM1-(X)
GH5_4
GH12
GH44
GH74
AA9
GH5_7
GH10
GH11
GH26
GH30_2
GH30_7
GH67
GH115
GH134
GH43
GH51
GH54
GH62
GH93
GH28
GH53
GH78
GH88
GH105
GH127
PL1
PL3
PL4
PL9
PL11
CE8
CE12

Supplementary Fig. 6. Transcriptional regulation of PCWDEs in the ectomycorrhizal ascomycete *Acephala macrosclerotiorum*. (**a**) A 3-week-old ectomycorrhizal rootlet of *Pinus sylvestris* colonized by *A. macrosclerotiorum* with typical black sclerotia formed on the surface of the rootlet (inset). The longitudinal section shows plant cell walls with nuclei (stained in red) and fungal cell wall chitin (stained in green) from colonizing hyphae forming a dense intraradicular network of mycelium. Note the absence of hyphal mantle on the surface of the rootlet. Confocal microscopy was carried out as described in [11]. (**b**) Heat map showing gene expression of key PCWDEs acting on cellulose, hemicellulose or pectin in ectomycorrhizal roots of *A. macrosclerotiorum* and other ectomycorrhizal species compared to their free-living mycelium. In white, no gene found in the genome; in yellow, gene(s) upregulated in ectomycorrhizal roots; in blue: gene(s) present in the genome, but not upregulated in ectomycorrhizal roots. Fungal species: Lacbi2, ectomycorrhizal *Laccaria bicolor*; Amamu1, ectomycorrhizal *Amanita muscaria*; Hebcy2, ectomycorrhizal *Hebeloma cylindrosporum*; Pilcr1, ectomycorrhizal *Piloderma croceum*; Paxin1, ectomycorrhizal *Paxillus involutus*; Tubma1, ectomycorrhizal *Tuber magnatum*; Cenge3, ectomycorrhizal *Cenococcum geophilum*; Acema1, ectomycorrhizal *Acephala macrosclerotiorum*; Melbi1, ericoid mycorrhizal *Meliniomyces bicolor*; Rhier1, ericoid mycorrhizal *Rhizoscyphus ericae*; Oidma1: ericoid mycorrhizal *Oidiodendron maius*. CAZymes gene expression data were extracted from previously published RNA-Seq experiments. Details can be found at the NCBI Gene Expression Omnibus in [12] for *L. bicolor*, *A. muscaria*, *H. cylindrosporum*, *P. croceum* and *P. involutus* (superseries GSE63947), in [13] for *T. magnatum* (GSE116692), in [14] for *C. geophilum (*GSE83909) and in [15] for *M. bicolor* (GSE107845*)*, *R. ericae* (GSE107647) and *O. maius* (GSE63922*)*. RNA-Seq data for *A. macrosclerotiorum* were submitted to NCBI-SRA with accession numbers SRP130276 and SRP130279-82. Source data are provided as a Source Data Supplementary figure 6.

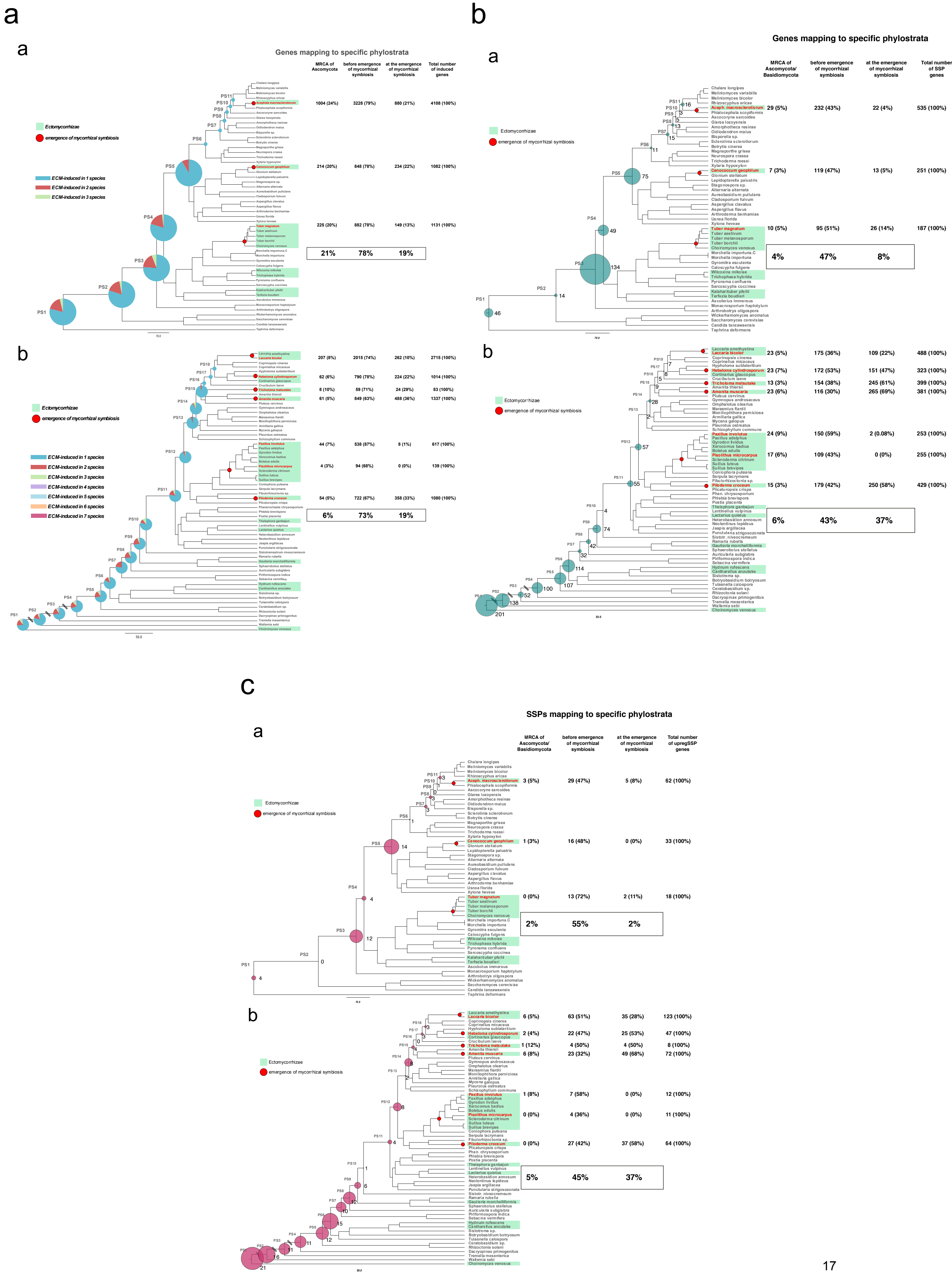

a
b
c
Genes mapping to specific phylostrata
SSPs mapping to specific phylostrata
MRCA of Ascomycota
MRCA of Ascomycota/ Basidiomycota
before emergence of mycorrhizal symbiosis
at the emergence of mycorrhizal symbiosis
Total number of induced genes
Total number of SSP genes
Total number of upregSSP genes
Ectomycorrhizae
emergence of mycorrhizal symbiosis
ECM-induced in 1 species
ECM-induced in 2 species
ECM-induced in 3 species
ECM-induced in 4 species
ECM-induced in 5 species
ECM-induced in 6 species
ECM-induced in 7 species
1004 (24%) 3228 (79%) 880 (21%) 4108 (100%)
214 (20%) 848 (78%) 234 (22%) 1082 (100%)
225 (20%) 882 (78%) 149 (13%) 1131 (100%)
21% 78% 19%
207 (8%) 2015 (74%) 262 (10%) 2715 (100%)
62 (6%) 790 (78%) 224 (22%) 1014 (100%)
8 (10%) 59 (71%) 24 (29%) 83 (100%)
61 (5%) 849 (63%) 488 (36%) 1337 (100%)
44 (7%) 538 (87%) 8 (1%) 617 (100%)
4 (3%) 94 (68%) 0 (0%) 139 (100%)
54 (5%) 722 (67%) 358 (33%) 1080 (100%)
6% 73% 19%
29 (5%) 232 (43%) 22 (4%) 535 (100%)
7 (3%) 119 (47%) 13 (5%) 251 (100%)
10 (5%) 95 (51%) 26 (14%) 187 (100%)
4% 47% 8%
23 (5%) 175 (36%) 109 (22%) 488 (100%)
23 (7%) 172 (53%) 151 (47%) 323 (100%)
13 (3%) 154 (38%) 245 (61%) 399 (100%)
23 (6%) 116 (30%) 265 (69%) 381 (100%)
24 (9%) 150 (59%) 2 (0.08%) 253 (100%)
17 (6%) 109 (43%) 0 (0%) 255 (100%)
15 (3%) 179 (42%) 250 (58%) 429 (100%)
6% 43% 37%
3 (5%) 29 (47%) 5 (8%) 62 (100%)
1 (3%) 16 (48%) 0 (0%) 33 (100%)
0 (0%) 13 (72%) 2 (11%) 18 (100%)
2% 55% 2%
6 (5%) 63 (51%) 35 (28%) 123 (100%)
2 (4%) 22 (47%) 25 (53%) 47 (100%)
1 (12%) 4 (50%) 4 (50%) 8 (100%)
6 (8%) 23 (32%) 49 (68%) 72 (100%)
1 (8%) 7 (58%) 0 (0%) 12 (100%)
0 (0%) 4 (36%) 0 (0%) 11 (100%)
0 (0%) 27 (42%) 37 (58%) 64 (100%)
5% 45% 37%

Supplementary Fig. 7. Phylostratigraphy for (a) ectomycorrhiza-specific upregulated genes, (b) genes coding for secreted proteins, and (c) genes coding for small secreted proteins (SSPs). Pie charts on the time-calibrated trees represent the number of gene clusters containing ectomycorrhiza-specific upregulated genes from the species compared. Numbers and percentage of genes mapping to phylostrata are shown on the right of the trees. Ectomycorrhizal species used for the comparison are in green boxes. Upregulated genes were selected according to FDR adjusted p-value < 0.05. Panels (**a**) and (**b**) display the upregulated genes (with fold-change ≥ 2) in Ascomycota and Basidiomycota, respectively. See Supplementary Data 9 for the identified phylostrata containing ectomycorrhiza-induced genes and related enrichment statistics. Source data are provided as a Source Data figure 4.

References

1. Grigoriev IV, Nikitin R, Haridas S, Kuo A, Ohm R, Otillar R, Riley R, Salamov A, Zhao X, Korzeniewski F, Smirnova T, Nordberg H, Dubchak I, Shabalov I. MycoCosm portal: gearing up for 1000 fungal genomes. *Nucleic Acids Res.* D699-704 (2014).

2. Kuo A, Bushnell B, Grigoriev IV. Fungal genomics: sequencing and annotation, p 1–52. In Martin F (ed), Fungi. Advances in botanical research. Elsevier Academic Press, Cambridge, United Kingdom. (2014).

3. Gnerre S, Maccallum I, Przybylski D, Ribeiro FJ, Burton JN, Walker BJ, Sharpe T, Hall G, Shea TP, Sykes S, Berlin AM, Aird D, Costello M, Daza R, Williams L, Nicol R, Gnirke A, Nusbaum C, Lander ES, Jaffe DB. High-quality draft assemblies of mammalian genomes from massively parallel sequence data. *Proc Natl Acad Sci USA*. **108**, 1513-1518 (2011).

4. Zerbino, D. R., Birney, E. Velvet: algorithms for de novo short read assembly using de Bruijn graphs. *Genome Res.* **18**, 821-829 (2008).

5. Chin CS, Peluso P, Sedlazeck FJ, Nattestad M, Concepcion GT, Clum A, Dunn C, O'Malley R, Figueroa-Balderas R, Morales-Cruz A, Cramer GR, Delledonne M, Luo C, Ecker JR, Cantu D, Rank DR, Schatz MC. Phased diploid genome assembly with single-molecule real-time sequencing. *Nature Methods* **13**, 1050-1054 (2016).

6. Koren S, Schatz MC, Walenz BP, Martin J, Howard JT, Ganapathy G, Wang Z, Rasko DA, McCombie WR, Jarvis ED, Adam M Phillippy. Hybrid error correction and de novo assembly of single-molecule sequencing reads. *Nature Biotechnol.* **30**, 693-700 (2012).

7. English AC, Richards S, Han Y, Wang M, Vee V, Qu J, Qin X, Muzny DM, Reid JG, Worley KC, Gibbs RA. Mind the gap: upgrading genomes with Pacific Biosciences RS long-read sequencing technology. *PLoS One* **7**, e47768 (2012).

8. Grabherr MG, Haas BJ, Yassour M, Levin JZ, Thompson DA, Amit I, Adiconis X, Fan L, Raychowdhury R, Zeng Q, Chen Z, Mauceli E, Hacohen N, Gnirke A, Rhind N, di Palma F, Birren BW, Nusbaum C, Lindblad-Toh K, Friedman N, Regev A. Full-length transcriptome assembly from RNA-Seq data without a reference genome. *Nature Biotechnol.* **29**, 644-652 (2011).

9. Martin, J., Bruno, V. M., Fang, Z., Meng, X., Blow, M., Zhang, T., Sherlock, G., Snyder, M., Wang, Z. Rnnotator: an automated de novo transcriptome assembly pipeline from stranded RNA-Seq reads. *BMC Genomics* **11**, 663 (2010).

10. Castanera, R., López-Varas, L., Borgognone, A., LaButti, K., Lapidus, A., Schmutz, J., *et al.* Transposable elements versus the fungal genome: Impact on whole-genome architecture and transcriptional profiles. *PLoS Genet.* **12**, e1006108 (2016).

11. Zhang, F. *et al.* The ectomycorrhizal basidiomycete *Laccaria bicolor* releases a secreted β-1,4 endoglucanase that plays a key role in symbiosis development. *New Phytol.*, **220**, 1309-1321 (2018).

12. Kohler, A. *et al.* Convergent losses of decay mechanisms and rapid turnover of symbiosis genes in mycorrhizal mutualists. *Nat. Genet.* **47**, 410–415 (2015).

13. Murat, C. *et al.* Pezizomycetes genomes reveal the molecular basis of ectomycorrhizal truffle lifestyle. *Nat Ecol Evol* **2,** 1956–1965 (2018).

14. Peter, M. *et al.* Ectomycorrhizal ecology is imprinted in the genome of the dominant symbiotic fungus *Cenococcum geophilum*. *Nat. Commun.* **7**, 12662 (2016).

15. Martino, E. *et al.* Comparative genomics and transcriptomics depict ericoid mycorrhizal fungi as versatile saprotrophs and plant mutualists. *New Phytol.* **217**, 1213–1229 (2018).
