# Supplementary material for: Large-scale genome sequencing of mycorrhizal fungi provides insights into the early evolution of symbiotic traits
Source: Nat Commun. 2020 Oct 12;11:5125. doi: 10.1038/s41467-020-18795-w (PMC7550596; doi:10.1038/s41467-020-18795-w)
Supplement: Supplementary file 3 — Reporting Summary [file 41467_2020_18795_MOESM3_ESM.pdf]

nature research

Corresponding author(s): Francis M. Martin

Last updated by author(s): 2020/09/03

Reporting Summary

Nature Research wishes to improve the reproducibility of the work that we publish. This form provides structure for consistency and transparency in reporting. For further information on Nature Research policies, see Authors & Referees and the Editorial Policy Checklist .

Statistics

For all statistical analyses, confirm that the following items are present in the figure legend, table legend, main text, or Methods section.

n/a	Confirmed	
☐	☒	The exact sample size (n) for each experimental group/condition, given as a discrete number and unit of measurement
☒	☐	A statement on whether measurements were taken from distinct samples or whether the same sample was measured repeatedly
☐	☒	The statistical test(s) used AND whether they are one- or two-sided<br>*Only common tests should be described solely by name; describe more complex techniques in the Methods section.*
☒	☐	A description of all covariates tested
☐	☒	A description of any assumptions or corrections, such as tests of normality and adjustment for multiple comparisons
☐	☒	A full description of the statistical parameters including central tendency (e.g. means) or other basic estimates (e.g. regression coefficient) AND variation (e.g. standard deviation) or associated estimates of uncertainty (e.g. confidence intervals)
☐	☒	For null hypothesis testing, the test statistic (e.g. F, t, r) with confidence intervals, effect sizes, degrees of freedom and P value noted<br>*Give P values as exact values whenever suitable.*
☒	☐	For Bayesian analysis, information on the choice of priors and Markov chain Monte Carlo settings
☒	☐	For hierarchical and complex designs, identification of the appropriate level for tests and full reporting of outcomes
☒	☐	Estimates of effect sizes (e.g. Cohen's d, Pearson's r), indicating how they were calculated

Our web collection on statistics for biologists contains articles on many of the points above.

Software and code

Policy information about availability of computer code

Data collection	Please refer to Supplementary Table 15 - List of software and R packages
Data analysis	Please refer to Supplementary Table 15 - List of software and R packages

For manuscripts utilizing custom algorithms or software that are central to the research but not yet described in published literature, software must be made available to editors/reviewers. We strongly encourage code deposition in a community repository (e.g. GitHub). See the Nature Research guidelines for submitting code & software for further information.

Data

Policy information about availability of data

All manuscripts must include a data availability statement. This statement should provide the following information, where applicable:

- Accession codes, unique identifiers, or web links for publicly available datasets
- A list of figures that have associated raw data
- A description of any restrictions on data availability

Genome assemblies and annotations for the organisms used in this study are available via the JGI fungal genome portal MycoCosm (mycocosm.jgi.doe.gov). In addition, the newly sequenced genome assemblies and annotations have been deposited to GenBank (see Supplementary Table 1 for accession codes/BioProjects). The complete transcriptome data sets are available at the Gene Expression Omnibus at the National Center for Biotechnology Information (http://www.ncbi.nlm.nih.gov/geo/). The accession codes for accessing the data deposited at Gene Expression Omnibus database are provided in the Methods/Phylostratigraphy section and in the caption of Supplementary Figure 6. All other data supporting the findings of this study are available within the article and its Supplementary Information files.

Field-specific reporting

Please select the one below that is the best fit for your research. If you are not sure, read the appropriate sections before making your selection.

- [ ] Life sciences
- [ ] Behavioural & social sciences
- [x] Ecological, evolutionary & environmental sciences

For a reference copy of the document with all sections, see nature.com/documents/nr-reporting-summary-flat.pdf

Ecological, evolutionary & environmental sciences study design

All studies must disclose on these points even when the disclosure is negative.

Study description	The study is a large-scale phylogenomic comparison of 135 recently sequenced fungal species. We predicted genes for secreted enzymes and counted the number of such genes for comparisons. Also, we constructed the ancestral state of genes using the organismal trees and transcriptomes of the extant species to assess the evolutionary dynamics of gene families.
Research sample	A total of 135 fungal genomes made of 73 saprotrophic, endophytic and pathogenic fungal species, and 62 mycorrhizal fungal species, including 29 new mycorrhizal genomes. This set samples major groups, for which there were previously no symbiotic genomes available, including Russulales, Thelephorales, Phallomycetidae, and Cantharellales. These groups are important, because they (1) are often ecologically dominant (Russulales and Thelephorales), (2) represent early-diverging clades for which no ectomycorrhizal genomes were previously available (Phallomycetidae and Cantharellales), and (3) include groups that arose before (Cantharellales) or after the origin of ligninolytic peroxidases (class II POD) implicated in white rot.
Sampling strategy	We gathered all the most recent JGI-sequenced genomes available for ectomycorrhizal fungi and evolutionarily related species. Then, comparative genomic analyses were performed by grouping 135 species according to their lifestyles/ecological traits.
Data collection	Please refer to: i) Supplementary Table 1 for the list of fungal species, the culture collection/collectors which/whom provided the fungal cultures; ii) Genomes were produced at the US DOE Joint Genome Institute following standardized protocols. Library constructions, genome sequencing platform and sequencing instruments used to produce the genomes and transcriptomes are described in the Supplementary Methods.
Timing and spatial scale	n/a - This is a phylogenomic study making use of fungal species which have been collected over decades in different countries. Information on the fungal cultures can be obtained from the culture collections.
Data exclusions	no data were excluded from the analyses
Reproducibility	All our data are available, which address the "reproducibility" issue. In addition, the Methods section, descriptions of statistical procedures, as well as the raw data provided should ensure that the analyses can be replicated.
Randomization	n/a - for the kinds of comparative genomic studies we performed there was nothing to be randomized.
Blinding	n/a - for the kinds of comparative genomic studies we performed there was nothing to be analyzed with blinding.

Did the study involve field work? [ ] Yes [x] No

Reporting for specific materials, systems and methods

We require information from authors about some types of materials, experimental systems and methods used in many studies. Here, indicate whether each material, system or method listed is relevant to your study. If you are not sure if a list item applies to your research, read the appropriate section before selecting a response.

Materials & experimental systems

n/a	Involved in the study	
x		Antibodies
x		Eukaryotic cell lines
x		Palaeontology
	x	Animals and other organisms
x		Human research participants
x		Clinical data

Methods

n/a	Involved in the study	
x		ChIP-seq
x		Flow cytometry
x		MRI-based neuroimaging

Animals and other organisms

Policy information about studies involving animals; ARRIVE guidelines recommended for reporting animal research

Laboratory animals	Fungal strains - no animals involved for our study.

Wild animals	n/a
Field-collected samples	n/a
Ethics oversight	n/a

Note that full information on the approval of the study protocol must also be provided in the manuscript.
