# Supplementary material for: Large-scale genome sequencing of mycorrhizal fungi provides insights into the early evolution of symbiotic traits
Source: Nat Commun. 2020 Oct 12;11:5125. doi: 10.1038/s41467-020-18795-w (PMC7550596; doi:10.1038/s41467-020-18795-w)
Supplement: Supplementary file 4 — Description of Additional Supplementary Files [file 41467_2020_18795_MOESM4_ESM.pdf]

Description of Additional Supplementary Files

Supplementary Data 1	Genomic features and descriptions of newly sequenced fungi
Supplementary Data 2	Taxonomic affiliation, genomic features and statistics for the 135 fungal genomes
Supplementary Data 3	Permutational multivariate analysis of variance (PERMANOVA) of genomic features
Supplementary Data 4	Identified transposable elements in the 135 fungal genomes
Supplementary Data 5	Count of orthologous genes in the clustered gene families of 112 fungal genomes
Supplementary Data 6	Count of proteomes and statistics for 135 fungi
Supplementary Data 7	Count of substrate-specific secretomes with statistics
Supplementary Data 8	Annotations and gene copy numbers of plant cell wall degrading enzymes (PCWDEs) used for COMPARE analysis
Supplementary Data 9	Summary of the phylostratigraphy analysis of symbiosis-induced genes of 10 ECM fungi
Supplementary Data 10	Occurrence in the genome and sequence similarity of ectomycorrhiza-upregulated genes from ten mycorrhizal fungi in the genome of the 135 fungi
Supplementary Data 11	CAZyme annotation reports
Supplementary Data 12	Small secreted proteins with known functions
Supplementary Data 13	Phylogenetic conservation of symbiosis-induced genes coding for secreted proteins among 135 fungi.
Supplementary Data 14	Phylogenetic conservation of symbiosis-induced genes coding for small secreted proteins among 135 fungi.
Supplementary	List of software and R packages

Data 15	
Supplementary Data 16	Comparison of gene expression from selected CAZy gene families in free-living mycelium and ectomycorrhizal root tips from *Acephala macrosclerotiorum*
