# Supplementary material for: Large-scale genome sequencing of mycorrhizal fungi provides insights into the early evolution of symbiotic traits
Source: Nat Commun. 2020 Oct 12;11:5125. doi: 10.1038/s41467-020-18795-w (PMC7550596; doi:10.1038/s41467-020-18795-w)
Supplement: Supplementary file 2 — Peer Review [file 41467_2020_18795_MOESM2_ESM.pdf]

Reviewers' comments:

Reviewer #1 (Remarks to the Author):

The manuscript by Miyauchi and co-workers presents a comparative analysis of 135 fungal genomes. 62 of these genomes are of mycorrhizal species and the authors have focused their analyses on the specific genomic signatures that define mycorrhizal lifestyle. The comparative genome analyses build on already published genomes as well as several new genomes described here for the first time. The authors describe a reduced set of lignin and cellulose degrading enzymes in mycorrhizal fungi as well as a large diversity of novel, lineage specific genes in mycorrhizal fungi. The authors suggest that these lineage specific genes encode secreted proteins that may function in the symbiotic interaction.

The dataset presented here provide an important contribution as it includes representative data from diverse groups of fungi from different phyla and from early diverging clades. Moreover, different types of mycorrhiza are represented including species that form arbuscular mycorrhiza, ectomycorrhiza, orchidmycorrhiza and ericoid mycorrhiza. With this impressive dataset the authors aim to address a fundamental question in fungal biology, namely which genomic traits determine mutualistic symbiosis with plant roots.

While the study presents an important genome resource to the community, I have several concerns regarding the analyses and the presentation of the results.

First of all, the introductory paragraph states "Our analyses support the general view that….". Hereafter the paragraph lists a number of genome characteristics, which have previously been associated with mycorrhizal lifestyle. In the discussion, the authors again highlight that their main findings are in agreement with already characterized patterns. Hereby, the novelty of the present study – besides including more genomes than previous studies – is hidden in the manuscript.

Furthermore, the authors do not clearly define a hypothesis. The manuscript has a strong focus on the metabolic capacity of mycorrhizal fungi and notably which traits that are reduced in the symbiotic species. Although the reduced saprothrophic capacity is important, the authors have to lesser extent emphasized the crucial acquisitions that allow the colonization and nutrient exchange with plants. In this context, an important hypothesis is that mycorrhizal symbiosis requires the production and secretion of effector molecules that can interfere with the plant immune system. While, the genome analyses include analyses of the gene repertoire encoding secreted proteins, this perspective is poorly considered throughout the manuscript. For example, the Introduction does not mention interaction with the plant immune system as a key determinant of mycorrhizal symbiosis. In my opinion this is an important point that should be put forward here as well as in the discussion.

The Glomerales and ericoid and orchid mycorrhiza are left out from several analyses, including the analysis of PCWDE as depicted in Figure 2. As mentioned in one line (182) the CAZyme content of the Glomerales is highly reduced. Nevertheless, it would be an interesting comparison to illustrate and outline as an example of an "extreme" adaptation to symbiosis (obligate symbiosis). I suggest highlighting stronger differences and similarities to this group of fungi as well as the ericoid and orhid mycorrhiza. The present dataset provides a unique opportunity to make such broad comparison.

The discussion almost exclusively focus on patterns in ectomycorrhiza. What do we learn about mycorrhiza origin for the other types of symbiosis? The discussion should embrace the entire dataset and the overarching objective of the study.

A main finding from comparing multiple mycorrhizal genomes is that genes encoding secreted proteins have diversified extensively and likely reflect adaptation to the plant-associated life-style. The manuscript presents a summary of the predicted secretome of the mycorrhizal fungi. This paragraph is relatively short compared to the putative importance of secreted proteins in the plant-fungus

interaction. I suggest including a more extensive analysis of the secretome. Important points to consider are:

- The prediction of effector proteins. A widely used approach to predict putative effector proteins in plant-associated fungi is the program EffectorP. EffectorP takes into account properties of proteins that are e.g. secreted into the apoplast of plant tissues. It is unclear how secreted proteins were annotated with the JGI pipeline. In my opinion the size criteria of 300 bp does not make sense and is not justified. Several effector proteins larger than 300 aa have been described in the literature. I would recommend amending the analyses with a specific effector prediction that takes size into account, but also other properties.

- In the assessment of convergent evolution, it would make sense to address similarities among predicted effector proteins. This is to some extent done as the supplementary results illustrate the extent of shared and unique SSPs. Can these analyses be taken further in terms of identifying shared effector repetoiores?

The authors have defined all 135 species according to a specific lifestyle (mycorrhiza, endophyte, wood decayer, saprotroph, pathogen). I assume that the biology of several of these fungi is poorly described. How many cases of "intermediate" categories or poorly defined lifestyles does the collection of fungi represent, and how did the authors handle these? The authors should comment on their criteria for ordering the fungi into the defined categories.

The manuscript has a strong focus on PCWDE. Obviously, the content and distribution of this class of enzymes provide an important difference between saprotrophs and mycorrhizal fungi. This finding is not novel. Nevertheless, the paragraph "Losses of plant cell wall degrading enzymes" is the longest paragraph in the manuscript. The paragraph moreover reads mostly like a long list where important details drown in un-necessary information. I note that the Ericoid mycorrhiza fungi, as a group, are not mentioned in this particular paragraph, although this group of fungi contains a large repertoire of PCWDEs. Overall, I suggest an extensive re-writing and shortening of this paragraph with a focus on some of the transition stages, new genomes and similarities across mycorrhizal types.

In the analysis of mycorrhizal induced genes (L. 283-293), it is relevant to test if genes encoding secreted proteins (effector candidates) are enriched compared to non-secreted. Furthermore, it is not clear why the analyses of evolutionary origins only include the ectomycorrhiza forming fungi? Is this due to a lack of RNAseq datasets?

Additional comments:

L 80: Please write out when used first time "Soil Organic Matter"

L. 123: "assembly" should be plural "assemblies"

L. 131-149: The title of the paragraph does not reflect the content of paragraph. This paragraph does not provide any details regarding birth and expansion of gene families. Please update paragraph titles to correctly report the main finding described.

L. 134-139: The listing of estimates of ranges of millions years can be moved to the supplementary text. Here it is sufficient to report the results obtained here and mention that these are younger than previously reported.

L. 160 and elsewhere throughout the manuscript where P-values are provided: Please mention in the parenthesis which test was used to compute the given P-value.

L. 193: What is meant with "total and secreted PCWDE"? Total loss and only loss of secreted

enzymes? Please rephrase.

L. 196-200: This information does not seem relevant for the crucial findings. I suggest to remove details like this to the supplementary text.

L. 282: The term "orphan" gene is also used as a gene that shows presence/absence variation within a species. I suggest only using the term "species-specific" here.

L. 416: Please write out WAG.

L. 424-432: This part can be moved to the supplementary text.

Methods in general: Scripts should also be made available.

Figure 4: The labels on the figure are mostly too small to be read. Even when zooming in on the pdf. Please change font size or format of the figure.

Suppl Fig. 1 , figure legend. I believe it should be "proportion" or "percentage " in stead of "Ratio"

Suppl Fig 2 and 4: Also here, I suggest using the term "proportion" in stead of "ratio" on the figure.

Suppl Fig 3, figure legend: Please mention the statistical test used when mentioning the P- value.

Reviewer #2 (Remarks to the Author):

Mycorrhizal Genomics Review

General impression

Miyauchi and coauthors report a large-scale comparative genomic analysis of mycorrhizal fungi, including a total of 135 fungal genomes of which 29 were newly sequenced mycorrhizal fungi. New sequences span a broad taxonomic range, including early diverging lineages and previously uncovered clades. The manuscript includes an overview of genome size and repeat content, secretomes, an in-depth evolutionary analysis of plant and fungal cell wall decomposition enzymes, and investigates the age distribution of genes active during symbiosis based on expression data from 10 species. The study is impressive in scope and will be of interest to the fungal genomics community, as well as evolutionary biologists studying convergent evolution. The methodology employed is state of the art in phylogenomics.

However, at the moment, the focus of the paper is largely confirmatory (gene loss, SSPs, CAZymes) and there are missed opportunities to emphasize the novel and use this new, more extensive dataset to go beyond what's been said in earlier publications.

In particular, conclusion #1: transitions involve gene losses -- has by now been the main conclusion of multiple publications. The conclusion is not novel. It's useful to have this science confirmed! But please consider editing the manuscript to emphasize what's new and leave this confirmatory conclusion as a side note, more or less.

Please see below for an elaboration of what we consider to be the most novel aspects of the manuscript:

Repeat content and genome size evolution analyses

This is a fantastic dataset to start employing the use of phylogenetic comparative methods to analyse dependence of genome size evolution and TE content on the phylogeny -- and to analyze whether these changes are coinciding with ecological shifts -- using a statistical framework. Previous comparative studies in this context were often too small for these types of analyses and these analyses would be a valuable and interesting addition to this paper and the discussion of the subject of genome architecture evolution in general. Not delving deeply into phylogenomic analyses of this sort is a missed opportunity.

Phylostratigraphy

This is a really novel, interesting approach. But for many origins of symbiosis, single lineages were sampled which complicates the message of species-specific genes and their importance in development of EcM symbioses. How can you tell if a gene is species-specific if only one species of the clade is sampled? Not surprisingly, this kind of sampling means the number of genes that are induced during symbiosis that arose at the time of symbiosis varies substantially among different origins, e.g. in the Boletales almost no genes induced were mapped to the origin of symbiosis. But is this just an effect of how many species were sampled within an origin of symbiosis, pushing the node deeper in time while there are still large numbers of species specific genes?

In general, without sampling more than one species, one cannot distinguish between genes that are genuinely species specific (e.g. if two species of a symbiotic lineage are sampled, each species has its own suite of genes induced) vs. genes that map to the origin of a symbiosis (e.g. if two species of a symbiotic lineage are sampled, the induced genes of both species are the same and map to the origin of symbiosis). We think that this is an important and really interesting distinction with a view towards evolutionary dynamics governing individual mycorrhizal lineages.

In the text there is talk about species-specific orphan genes which is used interchangeably with the category "Genes mapped to PS at the emergence of mycorrhizal symbiosis" but that equivalence is not 100% accurate. Do the clades where many EcM species sharing an origin also contribute to the "species-specific orphan" count? From the averages it appears so, so I think the wording in the text and interpretation of these metrics needs to be changed.

Distinguishing between clades like Laccaria, where more than one species IS sampled, vs. those where only one species is sampled, may be useful; so would taking more explicit advantage of the clades like Laccaria: these deeper clades provide an interesting opportunity to dissect the age distribution of EcM induced genes at finer granularity to get a better understanding of the impact of truly species-specific orphan genes as opposed to undersampling artefacts since each origin covers tens of millions of years. Perhaps an additional column in Fig. 4 could address this? Or an inset showing age distribution of EcM induced genes within the Boletales, Tuberaceae, Laccaria spp.?

The functional analysis of genes recruited at different phylogenetic distances is not based on the phylostratigraphy, but a clustering based on % identity of genes upregulated in all EcM species. This provides a different viewpoint than looking at shared recruitment from the same gene families in the phylogenomic data which would be more direct and informative for understanding which types of genes / families become repeatedly coopted and at what phylogenetic distance. Since the overlap is small, this would allow for an in-depth annotation of the gene sets in question.

Statistics

Often, statements are made without backing statistics, but with support from visuals. For example, Fig 1b/Fig 1c are used to suggest genome size is correlated with repeat element coverage. Box plots are presented, but if the data are being used to say e.g. line 122 "the main driver of genome inflation appeared to be repeat content", can statistics be used to explicitly test for a correlation? We're aware

that straight statistics e.g. a Pearson's correlation coefficient would be inappropriate, given the phylogenetic structure of the data, but phylogenetic comparative methods (see above) could be used to support these and other observations.

Detailed comments

Fig. 4 is very difficult to read. Are the two different threshold levels forcibly required or could one set move to the supplement since the patterns are overall similar.

L253 "thight"

Typo - should probably be tight

L433+
Two species trees / phylogenies were estimated and I'm wondering why the one including all species was not trimmed down to yield the other? Is there a specific reasoning or just that the analyses were run in parallel in different labs? Were there differences between the two approaches?

L505: which library of reference genes was used for BUSCO? Across all Eukaryotes? Fungi?

Data availability

Scripts should be posted to Github or another repository at the time of publication to ensure adequate access.

Similarly, I think files matching sequence IDs to cluster IDs should be made publicly available (e.g. via Dryad) to allow the community to properly make use and investigate the data generated in this study.

Manuscript NCOMMS-20-02957-T.

Large-scale genome sequencing of mycorrhizal fungi provides insights into the early evolution of symbiotic traits

Reviewers' comments:

Reviewer #1 (Remarks to the Author):

The dataset presented here provide an important contribution as it includes representative data from diverse groups of fungi from different phyla and from early diverging clades. Moreover, different types of mycorrhiza are represented including species that form arbuscular mycorrhiza, ectomycorrhiza, orchid mycorrhiza and ericoid mycorrhiza. With this impressive dataset the authors aim to address a fundamental question in fungal biology, namely which genomic traits determine mutualistic symbiosis with plant roots.

Authors. *Thanks for these positive comments.*

While the study presents an important genome resource to the community, I have several concerns regarding the analyses and the presentation of the results. First of all, the introductory paragraph states "Our analyses support the general view that….". Hereafter the paragraph lists a number of genome characteristics, which have previously been associated with mycorrhizal lifestyle. In the discussion, the authors again highlight that their main findings are in agreement with already characterized patterns. Hereby, the novelty of the present study – besides including more genomes than previous studies – is hidden in the manuscript.

Authors. *We agree that our general finding that ectomycorrhizal species have reduced complements of plant cell wall degrading enzymes compared to saprotrophs has been highlighted in prior publications. However, there is, we believe significant novelty in several regards. First, we have provided the first genomes for several major early diverging clades of ectomycorrhizal species for which there were no data. Second, there is considerable variation in the complements of plant cell wall degrading enzymes (PCWDEs) among mycorrhizal lineages. For example, the two mycorrhizal species of Phallomycetidae in our study,* Gautieria morchelliformis *and* Hysterangium stoloniferum, *have high numbers of PCWDEs, including ligninolytic class II peroxidases, which is unusual in this ecological guild. Within Cantharellales, the two newly sequenced ectomycorrhizal species,* Hydnum rufescens *and* Cantharellus anzutake, *are noteworthy because they deviate significantly from orchid symbionts in that clade, in terms of PCWDEs. In addition, we showed for the first time that transcriptional repression of PCWDEs is taking place in ectomycorrhizal species having a high repertoire of PCWDEs, i.e.* Acephala macrosclerotium. *Thus, we think that there is novelty here. We have endeavored to revise the ms, particularly the section "Losses of plant cell wall degrading enzymes", to emphasize what is new. This section has been extensively revised.*

We have added the following sentence in the Introduction section to make it clear: 'This dataset presents an opportunity to carry out a broader analysis of evolution of saprotrophic capabilities than that previously attempted by Kohler et al. (2015), who mainly focused on Agaricomycetidae.'

Furthermore, the authors do not clearly define a hypothesis.

Authors. *We respectfully disagree with the reviewer as we clearly stated our main aim(s) in the Abstract: "This study samples ecologically dominant fungal guilds for which there were previously no symbiotic genomes available, including Russulales, Thelephorales and Cantharellales … [to investigate] the transitions from saprotrophy to ectomycorrhizal symbiosis" and Introduction section: "To assess gains of ectomycorrhizal lifestyles, we discuss the fundamental adaptations that underlie convergent evolution of ectomycorrhizal fungi, including the loss of some metabolic functions, such as PCWDEs, and the acquisition of small secreted effector-like proteins that may facilitate the accommodation of symbiotic fungi within their host plants". In addition, we now clearly state in the last paragraph of the Introduction section: "We hypothesize that genomic traits determine mutualistic symbiosis with plant roots throughout the tree of life of Fungi."*

The manuscript has a strong focus on the metabolic capacity of mycorrhizal fungi and notably which traits that are reduced in the symbiotic species. Although the reduced saprothrophic capacity is important, the authors have to lesser extent emphasized the crucial acquisitions that allow the colonization and nutrient exchange with plants.

Authors. *In the revised manuscript, we included new comparative analyses of the main PFAM protein domains, transcription factors and membrane transporters throughout the 135 genomes to detect any symbiosis-related specificies (see heats maps showing the presence and abundance of the different Pfam domain-containing proteins, transcription factors and membrane transporters) in Supplementary figure 2). In contrast to the distribution of secreted PCWDEs (the trait explaining saprotrophic ability), no clear pattern in gene content can be observed for nutrient exchange or nutrient metabolism. These symbiosis-related acquisitions are controlled by the gene copy number of the saprotrophic vs. symbiotic genomes but through the transcriptomic regulation of their expression as shown previously in many studies (see papers by Tunlid's group on Paxillus involtus). However, we show here that the carbon flux from the host plant to the fungus in Basidiomycota is controlled by the apoplastic invertase of the plant partner as ectomycorrhizal species are missing the gene(s) coding for the secreted GH32 invertase.*

In this context, an important hypothesis is that mycorrhizal symbiosis requires the production and secretion of effector molecules that can interfere with the plant immune system. While, the genome analyses include analyses of the gene repertoire encoding secreted proteins, this perspective is poorly considered throughout the manuscript. For example, the Introduction does not mention interaction with the plant immune system as a key determinant of mycorrhizal symbiosis. In my opinion this is an important point that should be put forward here as well as in the discussion.

Authors. *We fully agree with the reviewer as we have published several research and review papers on the effector-like mycorrhizal-induced small secreted proteins (MiSSPs) and emphasized their key role in symbiosis establishment and mycorrhiza evolution. Our aim with the current paper was to focus on the evolution of the decay apparatus, but we also mentioned the effector-like SSPs in the phylostratigraphic analysis. In the revised version, we have added a few sentences in the Introduction and Discussion to emphasize the importance of the effector-like small secreted proteins (SSPs) in ectomycorrhizal development and establishment. We have also carried out additional analyses to document the genomic distribution of conserved, core SSPs and species-specific SSPs (see also Fig. 5 and Supplementary Fig. 7), throughout the 135 genomes We confirmed the main results of the*

phylostratigraphic study: most of the symbiosis-regulated SSPs are species-specific, although a few conserved SSPs, such as hydrophobins and lectins, have been co-opted during the symbiosis evolution to play structural roles..

The Glomerales and ericoid and orchid mycorrhiza are left out from several analyses, including the analysis of PCWDE as depicted in Figure 2. As mentioned in one line (182) the CAZyme content of the Glomerales is highly reduced. Nevertheless, it would be an interesting comparison to illustrate and outline as an example of an "extreme" adaptation to symbiosis (obligate symbiosis). I suggest highlighting stronger differences and similarities to this group of fungi as well as the ericoid and orchid mycorrhiza. The present dataset provides a unique opportunity to make such broad comparison.
Authors. *We have added a paragraph in the Discussion section to discuss the extremely low content of CAZYmes in Glomerales and the strikingly high repertoire of CAZymes in orchid and ericoid fungal symbionts. We are reluctant to discuss this topic in more detail as our analysis of the genomes of Glomeromycotina and ericoid fungi (including CAZymes) have been published in Martino et al. (2018) [Comparative genomics and transcriptomics depict ericoid mycorrhizal fungi as versatile saprotrophs and plant mutualists. New Phytologist doi: 10.1111/nph.14974] and Morin et al. (2019) Comparative genomics of Rhizophagus irregularis, R. cerebriforme, R. diaphanus and Gigaspora rosea highlights specific genetic features in Glomeromycotina. New Phytologist **222**: 1584-1598].*

The discussion almost exclusively focus on patterns in ectomycorrhiza. What do we learn about mycorrhiza origin for the other types of symbiosis? The discussion should embrace the entire dataset and the overarching objective of the study.
Authors. *As mentioned above, the evolution of arbuscular and ericoid mycorrhizas have been fiscussed in Martino et al. (2018) and Morin et al. (2019. However, we added a new paragraph in the Discussion section discussing discussing the entire set of genomes and the evolution of the different types of mycorrhizal symbioses.*

A main finding from comparing multiple mycorrhizal genomes is that genes encoding secreted proteins have diversified extensively and likely reflect adaptation to the plant-associated life-style. The manuscript presents a summary of the predicted secretome of the mycorrhizal fungi. This paragraph is relatively short compared to the putative importance of secreted proteins in the plant-fungus interaction. I suggest including a more extensive analysis of the secretome. Important points to consider are:
- The prediction of effector proteins. A widely used approach to predict putative effector proteins in plant-associated fungi is the program EffectorP. EffectorP takes into account properties of proteins that are e.g. secreted into the apoplast of plant tissues. It is unclear how secreted proteins were annotated with the JGI pipeline. In my opinion the size criteria of 300 bp does not make sense and is not justified. Several effector proteins larger than 300 aa have been described in the literature. I would recommend amending the analyses with a specific effector prediction that takes size into account, but also other properties.
Authors. *We have not used the JGI pipeline to annotate the secreted proteins. We have used a dedicated pipeline described in Pellegrin et al. (2015) Comparative analysis of secretomes from ectomycorrhizal fungi with an emphasis on small-secreted proteins. Front. Microbiol. 6. Prediction*

uses combined characteristics: proteins with a signal peptide as detected by SignalP v4.1 and no transmembrane domain or one overlapping signal peptide found, no internal localization (no endoplasmic reticulum addressing "KDEL" motif, secretory pathway by TargetP v1.1 and extracellular by WoLF PSORT 0.2) (see figure below).

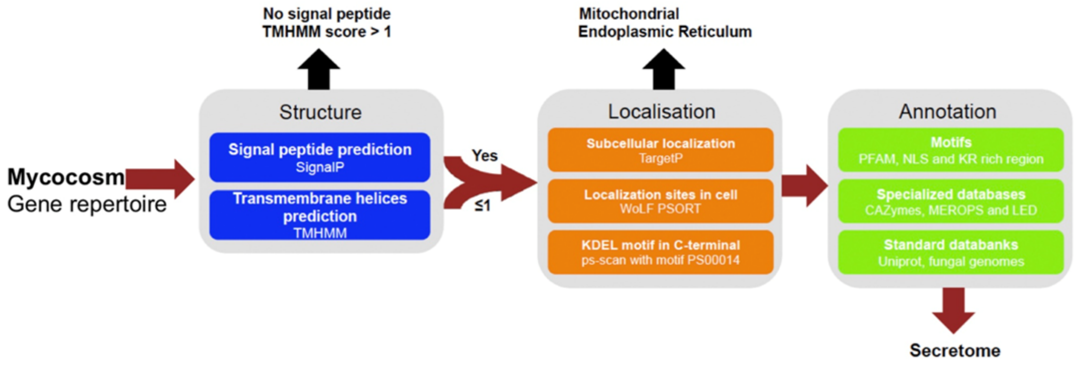

FIGURE 1 | Pipeline used to identify and annotate fungal secretome. Secreted proteins have been predicted based on genomic sequences from 49 fungal genomes retrieved from Joint Genome Institute (JGI, url: http://genome.jgi.doe.gov/Mycorrhizal_fungi/Mycorrhizal_fungi.info.html). Prediction uses combined characteristics: proteins with signal-peptide as detected by SignalP v4.1 and no transmembrane domain or one overlapping signal peptide founded, no internal localization (no endoplasmic reticulum addressing "KDEL" motif, secretory pathway by TargetP v1.1 and extracellular by WoLF PSORT 0.2). Proteins have been annotated according to motives scanning (PFAM, NLS, KR rich region), homologies found in specialized databases (CAZy database, MEROPS for proteases and Lipase Engineering Databases) and standard databanks (Uniprot, Swissprot, Mycocosm). SignalP4.1 is set to ≪ sensitive mode ≫, the other softwares are set with default parameters.

We have calibrated our in house pipeline by using known MiSSPs, such as MiSSP7, MiSSP7.6, and MiSSP8 (our publications), and showed that this pipeline outperformed EffectorP. EffectorP is not able to detect most of the MiSSPs we have/are characterized. EffectorP predicts effectors primarily by homology - this works in the Ascomycota where multiple plant pathogens' effectors have been experimentally characterized. We had mixed feelings about EffectorP, basically finding only hydrophobins (which are questionaly effectors) and a few others.

The size criteria of 300 bp has been used my most scientists working on effector-like proteins (also called candidate effector proteins) released by pathogenic and symbiotic filamentous microbes. Its true that the size cut-off of 300 bp is arbitratry, but we must conclude that it works. >90% of the functionally characterized effectors are <300 bp or ~100AA. This being said, the referee will be pleased to hear that we have also analyzed the secreted proteins having a size >300 bp with no known function. In our Supplemental figure 8, this gene category is included in the secreted proteins..

- In the assessment of convergent evolution, it would make sense to address similarities among predicted effector proteins. This is to some extent done as the supplementary results illustrate the extent of shared and unique SSPs. Can these analyses be taken further in terms of identifying shared effector repertoires?

Authors. *The Reviewer is likely aware that most effectors used by plant pathogenic and symbiotic filamentous microbes, but also phytopathogenic insects and nematodes, are highly specific and rarely conserved. In the revision, we have included a new BLASTP analysis of the sequence conservation/divergence of the secreted symbiosis-induced proteins identified in all available ectomycorrhiza transcriptome (i.e., 1028 genes). We display in Figure 6 the sequence similarity of these 1028 genes among the 135 analyzed fungal genomes and showed that most MiSSPs belong to*

specific-specific gene clusters (e.g., clusters V, VI and VII) recapitulating our studies of MiSSPs in ectomycorrhizal symbiosis. The repertoires of symbiosis-regulated SSPs is not conserved amongst symbiotic fungi. These genes have evolved de novo or they could have diverged so much that similarity to homologous sequences from saprotrophic ancestors cannot be detected. We added a paragraph to discuss these results.

The authors have defined all 135 species according to a specific lifestyle (mycorrhiza, endophyte, wood decayer, saprotroph, pathogen). I assume that the biology of several of these fungi is poorly described. How many cases of "intermediate" categories or poorly defined lifestyles does the collection of fungi represent, and how did the authors handle these? The authors should comment on their criteria for ordering the fungi into the defined categories.

Authors. *Attribution of a specific lifestyle to fungi is indeed challenging. However, the definition of the lifestyle of the ectomycorrhizal fungi is based on (1) a thorough analysis of the available literature, including recent lists of mycorrhizal fungi curated by experts in the field (e.g. Dr. Leho Tedersoo, Mark Brundrett et al. Ref. 5, 36), (2) the FunGuild database (Nguyen et al. (2016)), (3) $^{15}N/^{13}C$ natural abundance ratios when available and (4) expertise of expert mycologists and the senior authors whom worked on ectomycorrhizal fungi or saprotrophic fungi for several decades. In the revision, we now comment on these criteria used for ordering our selection into defined lifestyle categories.*

Ref. Nguyen NH, Song Z, Bates ST, Branco, S, Tedersoo L, Menke J, Schilling JS, Kennedy PG. 2016. FUNGuild: an open annotation tool for parsing fungal community datasets by ecological guild. Fungal Ecology 20:241-248.

The manuscript has a strong focus on PCWDE. Obviously, the content and distribution of this class of enzymes provide an important difference between saprotrophs and mycorrhizal fungi. This finding is not novel. Nevertheless, the paragraph "Losses of plant cell wall degrading enzymes" is the longest paragraph in the manuscript. The paragraph moreover reads mostly like a long list where important details drown in un-necessary information. I note that the Ericoid mycorrhiza fungi, as a group, are not mentioned in this particular paragraph, although this group of fungi contains a large repertoire of PCWDEs. Overall, I suggest an extensive re-writing and shortening of this paragraph with a focus on some of the transition stages, new genomes and similarities across mycorrhizal types.

Authors. *We have extensively revised this entire section in accordance with the reviewer's comments. We have tried to emphasize what is truly novel, and highlight similarities as well as differences between mycorrhizal genomes. The section has been reduced by over 30% in length.*

In the analysis of mycorrhizal induced genes (L. 283-293), it is relevant to test if genes encoding secreted proteins (effector candidates) are enriched compared to non-secreted. Furthermore, it is not clear why the analyses of evolutionary origins only include the ectomycorrhiza forming fungi? Is this due to a lack of RNAseq datasets?

Authors. *We performed a PERMANOVA analysis of SSPs and other secreted proteins (proteases, lipases, phosphatases, CAZymes) (Supplementary figure 3c). As (1) the development and functioning of arbuscular mycorrhizal symbiosis, ericoid mycorrhizal symbiosis and ectomycorrhizal symbiosis are very different in term of biological and morphological features and (2) most MiSSPs are lineage-*

specific as showed in Supplementary Figure 6. This being said, we have found conserved symbiosis-upregulated genes coding for small secreted proteins. They code for hydrophobins or secreted CAZymes as mentioned in the text and in Supplementary Figures and Tables.

Additional comments:

L 80: Please write out when used first time "Soil Organic Matter"

Authors. *Done*.

L. 123: "assembly" should be plural "assemblies"

Authors. *Done*

L. 131-149: The title of the paragraph does not reflect the content of paragraph. This paragraph does not provide any details regarding birth and expansion of gene families. Please update paragraph titles to correctly report the main finding described.

Authors. *Done.*

L. 134-139: The listing of estimates of ranges of millions years can be moved to the supplementary text. Here it is sufficient to report the results obtained here and mention that these are younger than previously reported.

Authors. *Done*

L. 160 and elsewhere throughout the manuscript where P-values are provided: Please mention in the parenthesis which test was used to compute the given P-value.

Authors. *Done*

L. 193: What is meant with "total and secreted PCWDE"? Total loss and only loss of secreted enzymes? Please rephrase.

Authors. *Done*

L. 196-200: This information does not seem relevant for the crucial findings. I suggest to remove details like this to the supplementary text.

Authors. *Done*

L. 282: The term "orphan" gene is also used as a gene that shows presence/absence variation within a species. I suggest only using the term "species-specific" here.

Authors. *We rephrased this part.*

L. 416: Please write out WAG.

Authors. *Done*

L. 424-432: This part can be moved to the supplementary text.
Authors. *We think this part is necessary to properly describe how organismal phylogenies were inferred. We kept it in the Main Text.*

Methods in general: Scripts should also be made available.
Authors. *Scripts have been deposited to GitHub.*
For the 'custom script' cited on line 419 (submitted pdf), we can cite a paper, see the text.

Figure 4: The labels on the figure are mostly too small to be read. Even when zooming in on the pdf. Please change font size or format of the figure.
Authors. *This figure has been changed for the sake of simplifcation. See below.*

Suppl Fig. 1 , figure legend. I believe it should be "proportion" or "percentage " in stead of "Ratio"
Authors. *Done.*

Suppl Fig 2 and 4: Also here, I suggest using the term "proportion" in stead of "ratio" on the figure.
Authors. *Done.*

Suppl Fig 3, figure legend: Please mention the statistical test used when mentioning the P-value.
Authors. *Done.*

Reviewer #2 (Remarks to the Author):

General impression
Miyauchi and coauthors report a large-scale comparative genomic analysis of mycorrhizal fungi, including a total of 135 fungal genomes of which 29 were newly sequenced mycorrhizal fungi. New sequences span a broad taxonomic range, including early diverging lineages and previously uncovered clades. The manuscript includes an overview of genome size and repeat content, secretomes, an in-depth evolutionary analysis of plant and fungal cell wall decomposition enzymes, and investigates the age distribution of genes active during symbiosis based on expression data from 10 species. The study is impressive in scope and will be of interest to the fungal genomics community, as well as evolutionary biologists studying convergent evolution. The methodology employed is state of the art in phylogenomics.

However, at the moment, the focus of the paper is largely confirmatory (gene loss, SSPs, CAZymes) and there are missed opportunities to emphasize the novel and use this new, more extensive dataset to go beyond what's been said in earlier publications.

In particular, conclusion #1: transitions involve gene losses -- has by now been the main conclusion of multiple publications. The conclusion is not novel. It's useful to have this science confirmed! But

please consider editing the manuscript to emphasize what's new and leave this confirmatory conclusion as a side note, more or less.

Authors. *We agree that our general finding that ectomycorrhizal species have reduced complements of plant cell wall degrading enzymes (PCWDEs) compared to saprotrophs has been highlighted in prior publications. However, there is, we believe significant novelty in several regards. First, we have provided the first genomes for several major clades of ectomycorrhizal species for which there were no data. Second, there is considerable variation in the complements of PCWDEs among mycorrhizal lineages. For example, the two mycorrhizal species of Phallomycetidae in our study,* Gautieria morchelliformis *and* Hysterangium stoloniferum, *have high numbers of PCWDEs, including ligninolytic class II peroxidases, which is unusual in this ecological guild. Within Cantharellales, the two newly sequenced ectomycorrhizal species,* Hydnum rufescens *and* Cantharellus anzutake, *are noteworthy because they deviate significantly from orchid symbionts in that clade, in terms of PCWDEs. Thus, we think that there is novelty here. We have endeavored to revise the ms, particularly the section "Losses of plant cell wall degrading enzymes", to emphasize what is new. This section has been extensively revised. Please see below for an elaboration of what we consider to be the most novel aspects of the manuscript.*

Repeat content and genome size evolution analyses

This is a fantastic dataset to start employing the use of phylogenetic comparative methods to analyse dependence of genome size evolution and TE content on the phylogeny -- and to analyze whether these changes are coinciding with ecological shifts -- using a statistical framework. Previous comparative studies in this context were often too small for these types of analyses and these analyses would be a valuable and interesting addition to this paper and the discussion of the subject of genome architecture evolution in general. Not delving deeply into phylogenomic analyses of this sort is a missed opportunity.

Authors. *In the revision, we have included new statistical analyses (PERMANOVA) to confirm that (1) the genome size is positively correlated to the increased content in transposable elements (TE) and (2) TE invasion/proliferation is coinciding with the ecological shift from saprotrophy to mycorrhizal symbiosis in all lineages investigated – i.e., the observed increased genome size is not related to the phylogeny but to the lifestyle (Supplementary Figure 3c).*

Regarding the phylogenomic analysis of TE, we carried out the following analyses:

– phylogenetic analysis of the most abundant, well conserved, LTR retrotransposons, i.e. Copia and Gypsy. We observed a very limited phylogenetic conservation between ectomycorrhizal clades, corroborating our previous analyses in Morin et al. (2019), Murat et al. (2018) and Kohler et al. (2015).

- macrosynteny analyses of several taxonomically-related genomes — genomes were either too distant or too fragmented by TE activities to provide useful information.

- we already dated the TE invasions in the Pezizomycetes genomes (Murat et al., 2018) and Glomeromycotina genomes papers (Morin et al., 2019). However, we included the age distribution of LTR retrotransposons in Agaricales in the present revision (Supplementary Figure 1b).

We showed that the number of retroelement copies in the different mycorrhizal species (even belonging to the same order) is highly variable (Figure 1C), indicating that invasions by different types of TE took place independently in different mycorrhizal fungi. As a result of massive TE proliferation, mycorrhizal genomes show a very high level of structural rearrangements, and macrosynteny analysis cannot be carried out to assess the impact of TE invasion(s) on the genome architecture/landscape.

We have added a paragraph in the Discussion section to summarize these findings.

This being said, we agree that our results do warrant a large-scale in-depth bioinformatics and evolutionary analysis across mycorrhizal genomes (and possibly other biotrophic fungi), which we hope to present in future work.

Phylostratigraphy

This is a really novel, interesting approach. But for many origins of symbiosis, single lineages were sampled which complicates the message of species-specific genes and their importance in development of EcM symbioses. How can you tell if a gene is species-specific if only one species of the clade is sampled? Not surprisingly, this kind of sampling means the number of genes that are induced during symbiosis that arose at the time of symbiosis varies substantially among different origins, e.g. in the Boletales almost no genes induced were mapped to the origin of symbiosis. But is this just an effect of how many species were sampled within an origin of symbiosis, pushing the node deeper in time while there are still large numbers of species specific genes?

In general, without sampling more than one species, one cannot distinguish between genes that are genuinely species specific (e.g. if two species of a symbiotic lineage are sampled, each species has its own suite of genes induced) vs. genes that map to the origin of a symbiosis (e.g. if two species of a symbiotic lineage are sampled, the induced genes of both species are the same and map to the origin of symbiosis). We think that this is an important and really interesting distinction with a view towards evolutionary dynamics governing individual mycorrhizal lineages.

In the text there is talk about species-specific orphan genes which is used interchangeably with the category "Genes mapped to PS at the emergence of mycorrhizal symbiosis" but that equivalence is not 100% accurate. Do the clades where many EcM species sharing an origin also contribute to the "species-specific orphan" count? From the averages it appears so, so I think the wording in the text and interpretation of these metrics needs to be changed.

Distinguishing between clades like Laccaria, where more than one species IS sampled, vs. those where only one species is sampled, may be useful; so would taking more explicit advantage of the clades like Laccaria: these deeper clades provide an interesting opportunity to dissect the age distribution of EcM induced genes at finer granularity to get a better understanding of the impact of truly species-specific orphan genes as opposed to undersampling artefacts since each origin covers tens of millions of years. Perhaps an additional column in Fig. 4 could address this? Or an inset showing age distribution of EcM induced genes within the Boletales, Tuberaceae, Laccaria spp.?

Authors. *In these analyses we were mostly interested in what is the extent of overlap between mycorrhiza-induced genes among the different lineages, not necessarily whether gene origins coincide with the origin of the mycorrhizal lifestyle. We agree that defining species-specific genes is difficult and that sampling more would be interesting, but we think that the main question on convergence was properly answered with this sampling as well.*

We revised the text to emphasize gene **-** *ECM origin coincidence only for clades, for which multiple species have been sampled and added information on this to the ms.*

The functional analysis of genes recruited at different phylogenetic distances is not based on the phylostratigraphy, but a clustering based on % identity of genes upregulated in all EcM species. This

provides a different viewpoint than looking at shared recruitment from the same gene families in the phylogenomic data which would be more direct and informative for understanding which types of genes / families become repeatedly coopted and at what phylogenetic distance. Since the overlap is small, this would allow for an in-depth annotation of the gene sets in question.
Authors. *We agree with the reviewer. These two independent approaches provide complementary results and confirm that your results are robust. In the revision,the phylostratigraphic analyses have been carried out to include secreted and small secreted proteins.*

Statistics
Often, statements are made without backing statistics, but with support from visuals. For example, Fig 1b/Fig 1c are used to suggest genome size is correlated with repeat element coverage. Box plots are presented, but if the data are being used to say e.g. line 122 "the main driver of genome inflation appeared to be repeat content", can statistics be used to explicitly test for a correlation? We're aware that straight statistics e.g. a Pearson's correlation coefficient would be inappropriate, given the phylogenetic structure of the data, but phylogenetic comparative methods (see above) could be used to support these and other observations.
Authors*. Agreed. In the revision, we implemented phylogeny-aware statistics for genomic features to support our statements. We revised the sections by adding statistical support from permutational multivariate analysis of variance (PERMANOVA) and the generalized least squares (GLS) procedure with the Brownian motion model for random evolution (see Supplementary Table 3).*

Detailed comments
Fig. 4 is very difficult to read. Are the two different threshold levels forcibly required or could one set move to the supplement since the patterns are overall similar.
Authors. *Agreed. We have kept only parts (a) and (c) and moved (b) and (d) to the Supplemental Figure.*

L253 "thight". Typo - should probably be tight.
Authors. *Done.*

L433+
Two species trees / phylogenies were estimated and I'm wondering why the one including all species was not trimmed down to yield the other? Is there a specific reasoning or just that the analyses were run in parallel in different labs? Were there differences between the two approaches?
Authors*. Asco- and Basidiomycota are too divergent to be included in single analysis. This would be a managable problem for the species tree, butit would make the comparative genomics, gene tree-based analyses untractable.*

L505: which library of reference genes was used for BUSCO? Across all Eukaryotes? Fungi?
Authors*. We used the Fungi dataset as indicated in M & M section.*

Data availability

Scripts should be posted to Github or another repository at the time of publication to ensure adequate access. Similarly, I think files matching sequence IDs to cluster IDs should be made publicly available (e.g. via Dryad) to allow the community to properly make use and investigate the data generated in this study.

Authors*. Scripts have been deposited to GitHub and files matching sequence IDs to cluster IDs have been deposited to the Dryad database.*

Reviewer #1 (Remarks to the Author):

The authors have replied to the concerns I raised to the previous version of the manuscript. Overall, my comments have all been addressed and I appreciate the new analyses and the modifications of the text.
I still have some minor comments to the presentation of the work. As mentioned previously, I think this is an important contribution to the field of fungal genomics. I understand it is a challenge to summarize a multitude of exciting and novel observations in a short manuscript. Nevertheless, I still think the Introduction needs some re-writing. The authors still have a strong focus on ectomycorrhiza in the Introduction, in fact half of the Introduction is turned around the biology of this particular group of symbionts. A novelty of this study is however the much broader comparison of symbiosis strategies. I think the outline of the study would stand much stronger in the Introduction if there would be a general focus on mutualistic symbiosis of fungi represented by the several types of mycorrhiza. Instead of starting with "Ectomycorrhizal fungi are mutualist species…." the manuscript could start with something like "Different types of mutualistic symbiosis between fungi and plants have evolved……." Also, I miss to see an outline of the specificities of orchid, ericoid and arbuscular mycorrhiza in the same way as they are carefully outlined for ectomycorrhiza.

L. 132, the genome dataset comprise species with different genome architecture in terms of ploidy. It would be good to make a statement that the comparisons of genomes are based on analyses of haploid genomes.

The authors have now clarified in the Materials and Methods how they defined "ecological guilds". However, in line 149 when the association of ecology and genome features is first outline, it is unclear what the term "ecology" covers here. Please include a line to say something like "we specified X number of ecological guilds and assessed the correlation of genome traits….."

L. 176: The difference in species-specific numbers is immense ranging from 994 to 39410. Please include a line what this exceptional number in Mycena galopus represent (repeat driven gene expansions?).

L. 346: For the broader readership is would be good to specify "symbiosis-induced" genes. Which specific stage does this exactly represent, early contact, first stage of mycorrhizal development?

Figure 4, I find this figure notably interesting in the context of the Suppl. Fig. 7 that shows the phylostrata analysis for the SSP genes. This figure underlines the importance of secreted proteins in the symbiosis evolution. If there would be a way to integrate the supplementary figure into the main manuscript, it would clearly illustrate that point.

Legend Fig. 5: Please specify what the clusters I-VII represent in the text.

Reviewer #2 (Remarks to the Author):

Overall I'm happy with the implemented changes. Especially the section on plant cell wall decomposition enzymes has improved and succeeds at better highlighting the novel aspects of the presented analyses.

Reviewer #3 (Remarks to the Author):

Miyauchi et al. present a comparative genomic study of ectomycorrhizal fungi. The authors apply phylostratigraphic approach in tracing the origin of symbiosis upregulated genes. To achieve this the

authors take Asco- and Basidiomycota consensus phylogeny to map the genes using orthogroups obtained by all-vs-all blast. In principle this strategy is valid, but there is some room for improvement. First, the authors should discuss the depth of their phylogenies in a way that explains why they use only fungal species to date the origin of orthologs. Fungal genes certainly have orthologs outside Holomycota. Moreover, why to analyze Asco- and Basidiomycota separately, as these groups for sure share some orthologous genes. I know that all-vs-all blast strategy could be computationally limiting and one way, and not the only one, to circumvent this problem is to restrict the number of species included in the analysis. In any case, this should be explained in the text. Second, the authors only report distribution of symbiosis upregulated genes over phylogeny. This is simply not enough as these numbers are only descriptive and do not bring per se evolutionary tendencies. However, the presented dataset allows for testing of the enrichment of symbiosis upregulated genes in specific phylostrata, and this could be easily done by hypergeometric test (or some other statistical test commonly used in enrichment problems). Idea would be to see if we should be surprised to see, for instance, many symbiosis upregulated genes at the emergence of mycorrhizal symbiosis, or the obtained numbers fit to the expected values and thus are not particularly evolutionary interesting. These procedures were already explained in the first phylostratigraphic paper that the authors cite, so I'm convinced that the authors could address my suggestions with minimal efforts.

NCOMMS-20-02957-A

Large-scale genome sequencing of mycorrhizal fungi provides insights into the early evolution of symbiotic traits

Response to the reviewer's comments

Reviewer #1

Reviewer. The authors have replied to the concerns I raised to the previous version of the manuscript. Overall, my comments have all been addressed and I appreciate the new analyses and the modifications of the text.

The Authors. Thank you.

Reviewer. I still have some minor comments to the presentation of the work. As mentioned previously, I think this is an important contribution to the field of fungal genomics. I understand it is a challenge to summarize a multitude of exciting and novel observations in a short manuscript. Nevertheless, I still think the Introduction needs some re-writing. The authors still have a strong focus on ectomycorrhiza in the Introduction, in fact half of the Introduction is turned around the biology of this particular group of symbionts. A novelty of this study is however the much broader comparison of symbiosis strategies. I think the outline of the study would stand much stronger in the Introduction if there would be a general focus on mutualistic symbiosis of fungi represented by the several types of mycorrhiza. Instead of starting with "Ectomycorrhizal fungi are mutualist species…." the manuscript could start with something like "Different types of mutualistic symbiosis between fungi and plants have evolved……" Also, I miss to see an outline of the specificities of orchid, ericoid and arbuscular mycorrhiza in the same way as they are carefully outlined for ectomycorrhiza.

The Authors. To comply with the reviewer's request (within the 5000 word limit), the Introduction has thoroughly been revised to introduce our broad comparison of mycorrhizal symbiosis strategies. We outlined the specificities of orchid, ericoid and arbuscular mycorrhiza briefly as they have been presented and discussed in our previous genome comparisons focusing on either the Glomeromycotina [Morin et al. (2019) *New Phytol.* **222**, 1584-1598) or ericoid mycorrhizal fungi [Martino et al. (2018) *New Phytol.* **217**, 1213–1229].
In addition, we referred the reader to a *Nature Review Microbiology* article published by Bonfante's group in July 2020 which outline unique and common traits in these major mycorrhizal symbioses. *Nat. Rev. Microbiol.* DOI: 10.1038/s41579-020-0402-3 (2020).

This being said, the 29 new mycorrhizal genomes are from ectomycorrhizal fungi and most of our analyses are dealing with the mode of nutrition of ectomycorrhizal fungi. Thus, we would like to keep a strong focus on this guild of fungi.

Reviewer. L. 132, the genome dataset comprise species with different genome architecture in terms of ploidy. It would be good to make a statement that the comparisons of genomes are based on analyses of haploid genomes.

The Authors. Done. See line 562

Reviewer. The authors have now clarified in the Materials and Methods how they defined "ecological guilds". However, in line 149 when the association of ecology and genome features is first outline, it is unclear what the term "ecology" covers here. Please include a line to say something like "we specified X number of ecological guilds and assessed the correlation of genome traits….."

The Authors. Done. See line 85-87. We also changed the term ecology to lifestyle to avoid any further confusion.

Reviewer. L. 176: The difference in species-specific numbers is immense ranging from 994 to 39410. Please include a line what this exceptional number in *Mycena galopus* represent (repeat driven gene expansions?).

The Authors. Done. We added the following sentence : *"The large number of species-specific genes in M. galopus is the result of several striking expansions of gene families. This will be discussed further in our forthcoming comparison of Mycenaceae genomes."*

Reviewer. L. 346: For the broader readership is would be good to specify "symbiosis-induced" genes. Which specific stage does this exactly represent, early contact, first stage of mycorrhizal development?

The Authors. Done. We added the following sentence line 246 : " *We examined the age distribution of genes induced at different stages of ectomycorrhiza establishment (early to late stages), so-called symbiosis-induced genes, by defining phylogenetic ages (phylostrata).*

We also added the following sentence on line 560: *"Note that this pooled set of ectomycorrhiza-induced genes represent a heterogeneous set of symbiosis-induced transcripts from ectomycorrhizal roots from different fungal-tree associations sampled at different stages of development, from early to late stage of symbiosis establishment."*

Reviewer. Figure 4, I find this figure notably interesting in the context of the Suppl. Fig. 7 that shows the phylostrata analysis for the SSP genes. This figure underlines the importance of secreted proteins in the symbiosis evolution. If there would be a way to integrate the supplementary figure into the main manuscript, it would clearly illustrate that point.

The Authors. If the manuscript length allowed, we can include this figure in the Main Text as a supplemental panel of Figure 4. We rely on the Editor decision.

Reviewer. Legend Fig. 5: Please specify what the clusters I-VII represent in the text.

The Authors. Done. We added the following sentence in the Figure 5 legend: "*Clusters I to VIII correspond to group of sequences sharing the same level of protein sequence similarity based on BlastP*."

Reviewer #2 (Remarks to the Author):

Overall I'm happy with the implemented changes. Especially the section on plant cell wall decomposition enzymes has improved and succeeds at better highlighting the novel aspects of the presented analyses.

The Authors. Thank you.

Reviewer #3 (Remarks to the Author):

Reviewer. Miyauchi et al. present a comparative genomic study of ectomycorrhizal fungi. The authors apply phylostratigraphic approach in tracing the origin of symbiosis upregulated genes. To achieve this the authors take Asco- and Basidiomycota consensus phylogeny to map the genes using orthogroups obtained by all-vs-all blast. In principle this strategy is valid, but there is some room for improvement. First, the authors should discuss the depth of their phylogenies in a way that explains why they use only fungal species to date the origin of orthologs. Fungal genes certainly have orthologs outside Holomycota.

The Authors. We opted for analyzing only fungal genomes to improve computational efficiency and because the origins of ECM are shallow evolutionary events, so we reasoned that additional resolution cannot be added by including non-fungal outgroup taxa.

Reviewer. Moreover, why to analyze Asco- and Basidiomycota separately, as these groups for sure share some orthologous genes. I know that all-vs-all blast strategy could be computationally limiting and one way, and not the only one, to circumvent this problem is to restrict the number of species included in the analysis. In any case, this should be explained in the text.

The Authors. We agree that Asco- and Basidiomycota share orthologous genes (see Fig. 5) and analyzing the two phyla together might have revealed even more ancient origins for some of the ectomycorrhiza-induced genes. We opted for analyzing a thoroughly selected subset of the sequenced genomes of either Asco- and Basidiomycota mainly for computational reasons. Still, the COMPARE analyses carried out on Asco- and Basidiomycota separately required heavy CPU allocation times. Running COMPARE on 135 fungi would have been prohibitively time consuming involving disproportionate costs.

We now explain this in the revision by adding a sentence at the end of the paragraph "*Phylostratigraphy*" in the M & M section.

Note that Figure 5, illustrating the phylogenetic conservation of ectomycorrhiza-induced genes among 135 fungi, provides additional, complementary, information on the conservation of ectomycorrhiza-related orthologous genes (via a BlastP analysis). For example, cluster V in Figure 5A showed ectomycorrhiza-induced genes conserved in the Glomeromycotina, Asco- and Basidiomycota, whereas clusters I, II and IV comprise ectomycorrhizal-induced genes conserved in both Asco- and Basidiomycota (Supplementary Tables 13 and 14).

Reviewer. Second, the authors only report distribution of symbiosis upregulated genes over phylogeny. This is simply not enough as these numbers are only descriptive and do not bring per se evolutionary tendencies. However, the presented dataset allows for testing of the enrichment of symbiosis upregulated genes in specific phylostrata, and this could be easily done by hypergeometric test (or some other statistical test commonly used in enrichment problems). Idea would be to see if we should be surprised to see, for instance, many symbiosis upregulated genes at the emergence of mycorrhizal symbiosis, or the obtained numbers fit to the expected values and thus are not particularly evolutionary interesting. These procedures were already explained in the first phylostratigraphic paper that the authors cite, so I'm convinced that the authors could address my suggestions with minimal efforts.

The Authors. We appreciate this suggestion. In the revision, we tested enrichment of ectomycorrhiza-induced genes via the Fisher's exact test in each of the phylostrata in both the Ascomycota and Basidiomycota. Consistent with our results, presented in the previous ms. (i.e., mapping of genes over the phylogeny), we did not find a clear trend for enrichment of genes in specific phylostrata. Some enrichment was observed in ancient and in terminal nodes, which is not surprising given the large number of ancient and orphan genes, but even this was not consistent among species. We therefore conclude that there is no remarkable period or node during the evolution of ectomycorrhizal

symbioses that contributed a significantly higher number of ectomycorrhiza-induced genes than expected based on genome-wide figures.

REVIEWERS' COMMENTS:

Reviewer #1 (Remarks to the Author):

The authors have carefully addressed the comments raised in my previous review. I am happy with the implemented changes.

Reviewer #3 (Remarks to the Author):

I have only one additional remark that relates to the following author's response:

"The Authors. We opted for analyzing only fungal genomes to improve computational efficiency and because the origins of ECM are shallow evolutionary events, so we reasoned that additional resolution cannot be added by including non-fungal outgroup taxa. "

The authors should expand on their explanation and add it in the text. It is critical for readers to understand the limits of the analysis and directions to improve it in the future.

NCOMMS-20-02957-B

Large-scale genome sequencing of mycorrhizal fungi provides insights into the early evolution of symbiotic traits

Response to the reviewer's comments

Reviewer #3 (Remarks to the Author):

Reviewer. Reviewer #3 (Remarks to the Author):
I have only one additional remark that relates to the following author's response: "The Authors. We opted for analyzing only fungal genomes to improve computational efficiency and because the origins of ECM are shallow evolutionary events, so we reasoned that additional resolution cannot be added by including non-fungal outgroup taxa. "
The authors should expand on their explanation and add it in the text. It is critical for readers to understand the limits of the analysis and directions to improve it in the future.

The Authors. We included the following paragraph in the M & M (lines 485-492):

[We opted for analyzing Ascomycota and Basidiomycota genomes separately, and only fungal genomes to improve computational efficiency and because the origins of the ectomycorrhizal symbioses are shallow evolutionary events, so we reasoned that additional resolution cannot be added by including non-fungal outgroup taxa. The computational step of similarity-based clustering, multiple sequence alignments and tree inference are particularly sensitive to the phylogenetic breadth that the species cover. Analyzing Ascomycota and Basidiomycota separately improved the accuracy of our predictions, by not having to analyze as deep nodes of the tree as the Dikarya (most recent common ancestor of Ascomycota and Basidiomycota).]
